# Electrochemical (Bio)Sensors for Antibiotic Residue Detection in Aquatic Animal Products: A Review

**DOI:** 10.3390/bios16070359

**Published:** 2026-06-28

**Authors:** Meiqing Yang, Qiuhe Hu, Suiping Wang, Haozi Lu, Song Liu

**Affiliations:** 1 Hunan Provincial Key Laboratory for Molecular Immunity Technology of Aquatic Animal Diseases, College of Life and Environmental Science, Hunan University of Arts and Science, Changde 415000, China; meiqingyang2012@163.com (M.Y.);; 2 School of Materials Science and Engineering, Hunan Institute of Technology, Hengyang 421002, China; 3 State Key Laboratory of Chemo and Biosensing, College of Chemistry and Chemical Engineering, Hunan University, Changsha 410082, China

**Keywords:** electrochemical (bio)sensors, antibiotic residues, aquatic animals, sample pretreatment, complex matrices

## Abstract

The rapid and sensitive quantification of antibiotic residues in aquatic animals is crucial for ensuring food safety and protecting public health. Electrochemical (bio)sensors show great potential in this field due to their quick response time, low cost, and ease of miniaturization. This paper presents a systematic review of advances in the electrochemical detection of eight classes of antibiotics: fluoroquinolones, sulfonamides, amphenicols, tetracyclines, nitrofurans, macrolides, aminoglycosides, and β-lactams in aquatic animal samples. It covers four types of sensors: direct electrochemical sensors, immunosensors, aptasensors, and molecularly imprinted sensors. The review emphasizes the electrochemical behavior of the targets, interface design, recognition elements, signal amplification strategies, and validation using real samples. It also summarizes the sample pretreatment methods for different classes of antibiotics. Finally, the paper analyzes key challenges related to adaptability to complex matrices, consistency in sample preparation, and validation with real-world samples. Additionally, it proposes future directions for development in this field.

## 1. Introduction

Food safety is essential to human health and public health, serving as a critical foundation for sustainable development. As the global population grows and demand for high-quality animal protein increases, aquatic foods are playing an increasingly significant role in the global food system. Besides being a source of high-quality protein, aquatic foods also provide various important micronutrients and offer unique benefits for enhancing dietary quality [1]. Meanwhile, the supply chain for farmed aquatic products is becoming more globalized, which means that their quality and safety are closely related to international trade, market access, and supply stability [2].

As aquaculture becomes more intensive and large-scale, issues related to the quality and safety of aquatic products are becoming increasingly significant. Research indicates that antimicrobial drugs are still widely used in aquaculture for disease prevention and control, and their usage patterns are closely linked to regulatory policies, farming practices, and environmental backgrounds in different countries and regions [3,4]. Improper use of antibiotics can result in residue accumulation in the edible tissues of aquatic animals, thereby posing food safety risks and potential health hazards to consumers [5]. Studies show that antibiotic residues in animal-derived foods can be associated with adverse health effects, including allergic reactions, immunotoxicity, and disruptions to the gut microbiota [6]. Furthermore, the use of antimicrobial drugs in aquaculture promotes the selection and spread of drug-resistant bacteria, which can cause lasting impacts on aquaculture ecosystems [7]. Environmental studies suggest that these risks can migrate and spread among the environment, animals, and human populations [8]. Therefore, antibiotic residues in aquatic products are not only a food safety concern but also closely related to public health risks within the “One Health” framework [8].

Under the current situation, accurately monitoring antibiotic residues in the edible tissues of aquatic animals is essential for protecting consumer health and ensuring compliance with trade regulations. Furthermore, it serves as a crucial foundation for conducting risk assessments related to antimicrobial resistance (AMR) and promoting the sustainable development of aquaculture. Currently, the detection of antibiotic residues primarily relies on two types of methods. The first are instrumental analytical techniques, most notably liquid chromatography–tandem mass spectrometry (LC–MS/MS). Due to its high sensitivity, good selectivity, and strong quantitative accuracy, this method remains the key approach for the confirmatory analysis of antibiotic residues [9]. However, these methods typically require complex sample preparation, specialized instruments, and skilled operators, which limits their application in rapid on-site screening and high-throughput preliminary screening. To improve their applicability in complex matrices, they are often combined with sample pretreatment techniques, such as QuEChERS and its derivative strategies [10,11]. Another class of methods consists of immunoassays, including enzyme-linked immunosorbent assays (ELISA) and lateral flow immunoassay strips. These methods are relatively simple to operate and suitable for rapid screening. However, in complex samples, they may be affected by factors such as matrix effects, cross-reactivity, and insufficient quantitative capability [12].

As a potential complementary analytical technique, electrochemical sensing offers advantages such as fast response times, low cost, ease of miniaturization, and simple device integration, along with high sensitivity and reliable quantitative performance [13,14]. As a result, it serves as a valuable complementary tool bridging laboratory-validated analysis and rapid on-site screening. On the one hand, strategies such as nanomaterial modification, signal amplification, and ratiometric designs can be employed to enhance the sensitivity and quantitative reliability of electrochemical sensors [15,16]. On the other hand, the introduction of recognition elements such as antibodies [17], aptamers [18], and molecularly imprinted polymers (MIPs) [19] significantly improves the molecular recognition capabilities of the sensing system. However, electrochemical sensors cannot completely avoid the analytical challenges posed by complex aquatic matrices. Proteins, lipids, salts, and endogenous small molecules in aquatic animal tissue extracts may interfere with electron transfer at the electrode interface, target recognition, and signal output, thereby affecting the sensor’s detection performance [20]. Therefore, developing appropriate sample pretreatment methods, constructing anti-contamination interfaces, and validating the technology with real samples are crucial for evaluating the practical application value of electrochemical sensors in the detection of antibiotic residues in aquatic products.

In recent years, research on the electrochemical detection of antibiotic residues in aquatic products has grown rapidly. However, existing reviews focus on either broad areas or specific topics. Some reviews address antibiotic biosensing technologies in animal-derived foods or broader food matrices, summarizing the applications of various sensing platforms, recognition elements, and signal amplification strategies [21,22]. Other reviews have explored advances in antibiotic biosensing platforms from the perspectives of food safety, environmental monitoring, and clinical diagnosis [23]. In addition, some reviews have specifically focused on biosensors for seafood safety control [24], electrochemical aptasensors for food quality and safety assessment [25], and enzyme-based biosensors for detecting antibiotic residues in aquaculture [26]. Recently, Ying et al. provided a systematic review of rapid on-site detection technologies for antibiotic residues in aquatic products from 2021 to 2025, covering methods such as fluorescence sensing, lateral flow immunoassays, surface-enhanced Raman scattering, enzyme-linked immunosorbent assays, electrochemical sensing, and colorimetric sensing [27]. Although existing studies have provided important references for the detection of antibiotic residues in aquatic products, there is still a lack of a comprehensive review specifically focused on electrochemical detection of antibiotic residues in aquatic products.

Compared to existing reviews, this paper focuses on complex aquatic matrices (e.g., aquatic animals and their edible tissues), particularly exploring the impact of matrix components (including proteins, lipids, salts, and endogenous small molecules) on electrode interfaces, recognition processes, and detection reliability. Additionally, this paper compares the applicability of traditional laboratory electrodes with portable platforms such as screen-printed electrodes (SPE), paper-based electrodes, laser-induced graphene electrodes (LIGE), and flexible electrodes. It specifically discusses sample pretreatment strategies for aquatic animals, emphasizing that pretreatment in electrochemical detection must strike a balance between target release, elimination of matrix interference, sensor interface compatibility, and signal stability. Based on the above characteristics, this paper systematically summarizes the research progress in the electrochemical detection of antibiotic residues in aquatic products, focusing on the electrochemical response characteristics, detection mechanisms, sensing interface design, sample pretreatment strategies, and validation with real samples for different classes of antibiotics. Finally, this paper analyzes the main challenges and future development directions in practical applications of this field.

## 2. Technical Foundations for Electrochemical Detection of Antibiotic Residues in Aquatic Animals

**Figure 1:**
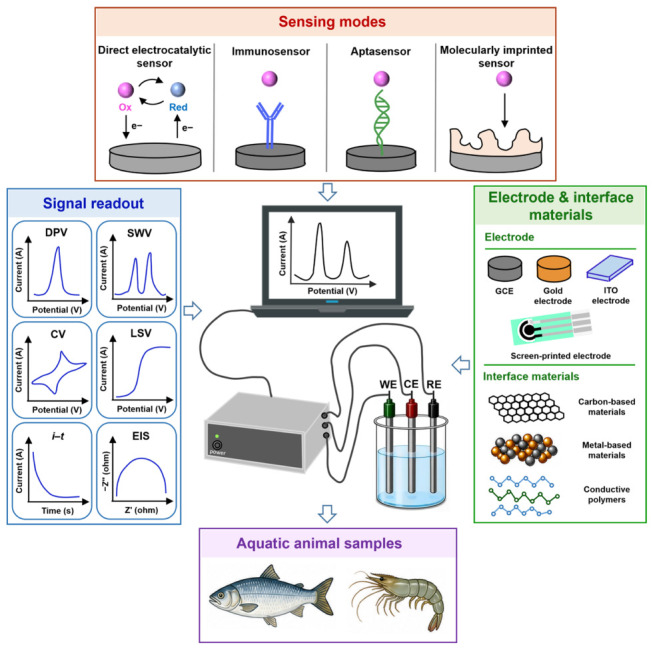
Technical foundations for electrochemical detection of antibiotic residues in aquatic animals.

Electrochemical detection of antibiotic residues in aquatic animal samples requires converting the intrinsic redox behavior of target molecules, or changes in recognition, adsorption, and charge transfer caused by them at the electrode interface, into measurable electrical signals. By rationally selecting electrode platforms, interface materials, recognition elements, and signal amplification strategies, electrochemical detection can achieve rapid analysis of trace antibiotic residues. Although different classes of antibiotics differ in molecular structure, electrochemical activity, and recognition requirements, their detection systems are generally built on a common technical foundation, mainly including signal readout, electrode platform and sensing interface construction, and recognition modes (Figure 1). Therefore, before discussing specific detection methods for different classes of antibiotics, it is necessary to outline the general principles of electrochemical detection and signal detection methods, the electrode platforms and strategies for sensing interface construction, as well as the recognition modes and signal enhancement methods.

### 2.1. Electrochemical Detection Principles and Common Signal Detection Methods

The basic principle of electrochemical detection is to convert the redox reactions, adsorption behaviors, recognition events, or charge transfer changes caused by target molecules at the electrode interface into measurable electrical signals. In antibiotic residue detection, the electrochemical response typically originates from two processes: one is the oxidation or reduction reaction of the target antibiotic itself at a specific potential, generating a peak current proportional to its concentration. The other is the binding of the target molecule with recognition elements such as antibodies, aptamers, or MIPs, thereby altering the steric hindrance, charge distribution, electron transfer efficiency, or diffusion process of the redox probe at the electrode interface, thus causing changes in the current or impedance signal [22,28]. The former is generally applicable to antibiotics with significant electroactivity, such as fluoroquinolones, tetracyclines, nitrofurans, and chloramphenicol. The latter is more suitable for targets with weaker electrochemical responses or requiring highly selective recognition.

Commonly used electrochemical methods in antibiotic residue detection include differential pulse voltammetry (DPV), square wave voltammetry (SWV), cyclic voltammetry (CV), linear sweep voltammetry (LSV), chronoamperometry (*i*–*t*), and electrochemical impedance spectroscopy (EIS) [28]. Among these, DPV and SWV are characterized by low background current, high signal-to-noise ratio, and good sensitivity and are commonly used for the quantitative analysis of trace antibiotic residues. CV is primarily used to analyze the redox behavior of target molecules, determine electrode reaction characteristics, and characterize the stepwise construction process of the sensing interface. LSV and *i*–*t* are frequently used for direct electrocatalytic detection or monitoring steady-state current responses. EIS reflects the construction of the recognition layer and the target binding process by monitoring changes in interfacial charge transfer resistance, making it particularly suitable for label-free immunosensors, aptasensors, and molecularly imprinted sensors. Different signal readout methods should be selected based on the electrochemical activity of the target antibiotic, the recognition mode, the interface material, and the complexity of the actual sample.

### 2.2. Electrode Platforms and Strategies for Constructing Sensing Interfaces

Electrode platforms and sensing interfaces are key factors influencing the sensitivity, selectivity, stability, and practical application potential of electrochemical sensors. Common working electrodes can be broadly categorized into two categories: one consists of traditional laboratory electrodes, such as glassy carbon electrodes (GCE), gold electrodes, and indium tin oxide (ITO) electrodes. These electrodes have advantages such as a wide electrochemical window, repeatable surface treatment, and controllable interface modification and are often used for mechanism research and the construction of high-performance sensing interfaces. The other category consists of disposable and portable electrode platforms, such as SPEs, paper-based electrodes, LIGEs and flexible electrodes. These platforms have the characteristics of low cost, easy mass production, small sample requirements, flexible design, and easy integration, and are more suitable for rapid screening and on-site detection in food safety applications [29,30]. With the development of portable electrochemical workstations, miniaturized detection devices, and integrated sensing platforms, the feasibility of applying electrochemical sensors in on-site analysis has been further enhanced [31]. Therefore, when evaluating electrochemical sensors for antibiotic detection, it is necessary not only to compare detection limits and linear ranges but also to analyze their applicable scenarios based on the characteristics of the electrode platform.

Regarding the construction of sensing interfaces, existing research primarily employs strategies such as conductivity enhancement, catalytic amplification, and immobilization of recognition elements to improve detection performance. Common electrode modification materials include carbon-based materials, metal-based materials, and conductive polymers. Carbon-based materials, such as carbon nanotubes, graphene and their derivatives, are typically used to enhance electrode conductivity, increase effective specific surface area, and facilitate electron transfer. Metal-based materials, such as metal nanoparticles and metal oxides, can enhance the redox reaction efficiency of the target antibiotics, thereby increasing response intensity and lowering detection limits [15,28]. Conductive polymers can simultaneously improve interfacial conductivity, film-forming properties, and the immobilization environment for biomolecules. For immunosensors, aptasensors, and molecularly imprinted sensors, interface construction also needs to provide stable immobilization sites for recognition elements. For example, antibodies, aptamers, or MIPs can be stabilized through Au-S bonds, EDC/NHS coupling, amino/carboxyl interactions, chitosan encapsulation, or polydopamine coatings [22,31]. Furthermore, since proteins, lipids, salts, and endogenous small molecules in aquatic animal samples can lead to electrode contamination and nonspecific adsorption, sensor interface design should also consider anti-contamination properties and adaptability to complex matrices. Therefore, sensor interface design not only involves the modification of electrode materials but also requires comprehensive consideration of electron transfer efficiency, electrocatalytic activity, immobilization of recognition elements, and adaptability to complex matrices.

### 2.3. Recognition Modes and Signal Enhancement Strategies

Electrochemical methods for detecting antibiotic residues in aquatic animal samples can generally be classified into two main modes: direct electrocatalytic detection and recognition-based detection. Direct electrocatalytic detection relies on the intrinsic electroactivity of the target molecule or the electrocatalytic reactions at the functional material interface to generate a response. It has the advantages of simple structure, rapid response, simple operation and low cost, and is suitable for targets with clear redox responses. On the other hand, recognition-based detection enhances selectivity by introducing specific recognition elements. This approach primarily includes immunosensing, aptasensing, and molecularly imprinted sensing, which are more suitable for trace analysis of targets with weak electrochemical responses or high selectivity requirements in complex sample systems [22]. Among these, antibodies and aptamers achieve specific recognition through antigen–antibody binding and aptamer–target binding, respectively, while MIPs achieve selective recognition by forming recognition sites in the polymer network that match the size, shape, and functional groups of the target molecule. For example, MIPs have been successfully used to selectively recognize enrofloxacin (ENR) and sulfadiazine (SDZ) in the detection of fluoroquinolones and sulfonamides [32,33].

To further improve detection performance, different detection modes typically require the combination of suitable interface materials and signal enhancement strategies. Research often employs methods such as nanomaterial modification, electrocatalytic sensitization, and enzymatic amplification to enhance response intensity and reduce detection limit [15]. It is important to note that while recognition elements improve selectivity, they also increase the manufacturing cost and application complexity of the sensor. Antibodies are generally expensive and sensitive to storage and handling conditions, which can easily increase the manufacturing and storage costs of immunosensors. Although aptamers are easier to synthesize and modify than antibodies, the screening of high-affinity aptamers, sequence modification, and surface immobilization still increase the cost of sensor construction. In contrast, MIPs generally have advantages such as lower preparation costs, better chemical stability, and the ability to replace certain biomolecular recognition elements. However, their recognition performance remains influenced by factors such as the uniformity of the imprinted sites, the integrity of the eluted template, and cross-reactivity with structural analogs. Therefore, in practical sensor design, it is necessary to comprehensively balance selectivity, sensitivity, cost, stability, and preparation complexity based on the electrochemical behavior of the target, the complexity of the sample matrix, and the application scenario.

## 3. Electrochemical Detection of Eight Classes of Antibiotics in Aquatic Animals

This section systematically summarizes research on the electrochemical detection of antibiotic residues in aquatic animal samples, focusing on the molecular structures, electrochemical response characteristics, primary detection strategies, analytical performance, and applicability to real-world samples of different antibiotic classes. For representative studies, this paper further analyzes the innovative aspects of their sensor designs and the key challenges they address, such as weak intrinsic response of the target, insufficient electron transfer efficiency, limited selectivity, interference from complex matrices, inadequate signal amplification efficiency, and limited suitability for on-site detection. Additionally, the last paragraph for each class of antibiotics summarizes the primary detection strategies, technical characteristics, and issues requiring further attention, highlighting both common patterns and specific challenges in the electrochemical detection of different antibiotic categories. Table 1 summarizes representative studies, sensor platforms, detection strategies, analytical performance, and applications in real samples for the electrochemical detection of different classes of antibiotics in aquatic animal samples.

It should be noted that the LODs listed in Table 1 do not mean that all detection methods must achieve extremely low LODs in practical applications. Existing research and relevant regulatory documents indicate that the actual detection requirements for antibiotic residues in aquatic animals should be determined in conjunction with the regulatory status and MRL of the specific analyte [70]. Meanwhile, there are differences in the MRLs and regulatory practices for antimicrobial drugs in aquatic products among different countries and regions [71]. Therefore, for antibiotics that are approved for use and have established MRLs, the sensor’s LOD typically meets rapid screening requirements when it is lower than the corresponding MRL. However, for prohibited antibiotics, such as chloramphenicol and nitrofurans, a lower LOD is critical for the identification and early warning of trace residues. Consequently, when evaluating the practical application potential of electrochemical sensors, one should not only compare LOD values but also comprehensively consider linear range, matrix effect, recovery rate, repeatability, verification with real samples, and comparison results with confirmatory methods.

### 3.1. Fluoroquinolones

Fluoroquinolones are one of the most extensively studied classes in the electrochemical detection of aquatic animal samples. ENR has been identified as a primary target, with other primary targets including norfloxacin (NOR), ciprofloxacin (CIP), and gatifloxacin (GAT). These antibiotics generally demonstrate notable intrinsic electrochemical activity, facilitating direct detection [72]. Mechanistic studies have shown that the electrochemical oxidation of fluoroquinolones mainly involves N-containing heterocyclic side chains, particularly piperazine or piperazine-like structures, providing a chemical basis for direct oxidation detection strategies [73]. However, co-extractable components and structurally similar analogs that may be present in extracts from fish, shrimp, and other biological tissues can affect the accuracy of quantitative measurements. Therefore, molecular imprinted and immunorecognition strategies have been widely developed for the detection of these antibiotics.

**Figure 2:**
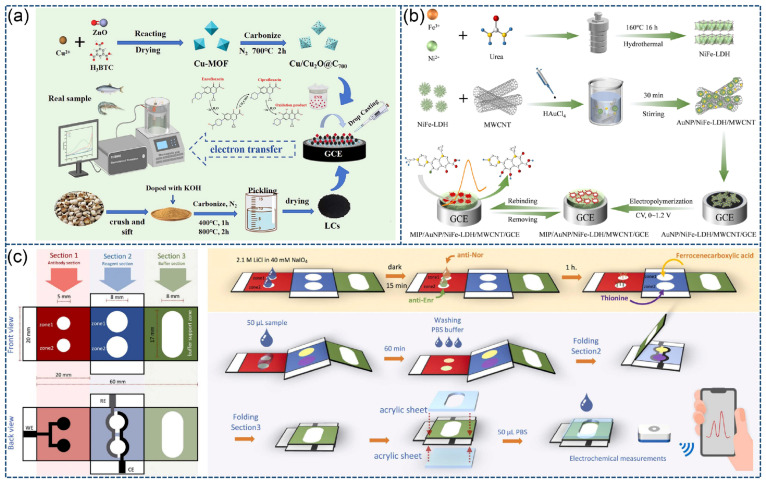
(**a**) Fabrication process of the Cu/Cu_2_O@C_700_/NCs/GCE-based sensor for the direct electrochemical detection of ENR. Reproduced with permission from [35]. (**b**) Preparation of the AuNP/NiFe-LDH/MWCNT/GCE-based molecularly imprinted sensor and its application in the detection of ENR. Reproduced with permission from [36]. (**c**) Composition of the label-free dual-channel electrochemical immunosensor on an origami-paper-based analytical device (**left**) and its detection process for NOR and ENR (**right**). Reproduced with permission from [38].

From a technical perspective, the electrochemical detection of fluoroquinolones can be classified into two main approaches: direct electrocatalytic detection and selective recognition. Regarding direct electrocatalytic detection, to address the weak ENR signal on traditional bare electrodes, Pervaiz et al. exploited the synergistic effect of Ce^3+^/Ce^4+^ redox properties and Mo-based active sites in Ce_2_(MoO_4_)_3_ to enhance the electrode response toward ENR without relying on antibodies or noble metals. This approach achieved a linear range of 0.1–12 µM and a LOD of 0.035 µM, with recovery rates of 98.7–101% (RSD < 1.8%) in fish, shrimp, and water samples. However, interference may occur in the presence of multiple antibiotics, and complex samples still require appropriate separation or purification [34]. To address the limited active sites and insufficient electron transfer efficiency in single-material systems, another study designed a composite interface consisting of Cu-MOF-derived porous carbon (Cu/Cu_2_O@C_700_) and nitrogen-doped longan shell carbon (NCs) for ENR detection (Figure 2a). In this system, Cu/Cu_2_O@C_700_ provides electrocatalytically active sites, while NCs enhance electron transport and ion diffusion. The synergistic effect of these two components reduced the detection limit for ENR to 7.73 × 10^−3^ ng/mL. The recovery rates in fish and shrimp samples ranged from 93.9% to 104.0% (RSD < 4%), and the results correlated well with those obtained from LC-MS/MS analysis [35]. In addition, systems such as reduced carbon nanotube (rCNT)/GCE, graphene-sodium polyacrylate-Pd (GN-PAAS-Pd)/GCE, reduced graphene oxide (rGO)/carbon paste electrode (CPE), and Ag_2_S/rGO/GCE have also been used for the direct detection of ENR, CIP, and GAT, respectively, further demonstrating the applicability of carbon-based materials and their composite interfaces with metal or semiconductor nanomaterials in enhancing the electrochemical response to fluoroquinolones [74,75,76,77]. Li et al. combined laser-induced graphene (LIG), poly(3,4-ethylenedioxythiophene) (PEDOT), and AuNPs to achieve simultaneous detection of chloramphenicol (CAP) and ENR through the synergistic catalytic effect of the multicomponent interface, which was validated in grass carp samples [78]. Furthermore, the Cu@Ag@CNTs/MWCNT system has expanded to enable simultaneous cross-category detection of NOR and isoproturon, indicating that direct electrochemical detection platforms are evolving from single-target analysis toward the combined detection of multiple contaminants [79].

In terms of selective recognition, molecularly imprinted sensing is one of the most mature methods. Chen et al. used multi-walled carbon nanotubes (MWCNTs) as a conductive support material, synthesized ENR MIPs via the sol–gel method, and coated them onto a GCE. This method utilizes MWCNTs to construct a conductive network, thereby enhancing interfacial electron transfer efficiency and effective contact area. Simultaneously, molecularly imprinted sites enhance the selective recognition of ENR, achieving a wide linear range of 2.8 pM to 28 µM and a low LOD of 0.9 pM, with recovery rates of 96.4–102% (RSD < 4.3%) in seawater, fish, and shrimp samples [32]. In order to overcome the limitations of traditional MIP-based sensors such as restricted interfacial charge transfer and insufficient active sites, Gu et al. designed a ternary heterostructure of AuNP/NiFe-LDH/MWCNT to modify a GCE. In this structure, MWCNTs serve as a conductive scaffold, NiFe-LDH nanosheets provide catalytic active sites, AuNPs facilitate interfacial charge transfer, and the ENR MIP layer is constructed via the electropolymerization of pyrrole (Figure 2b). This composite interface achieves synergistic signal enhancement and selective recognition, enabling the sensor to exhibit a linear response in the range of 0.05–100.0 µM with a LOD of 0.025 µM and recovery rates of 94.6–103.9% in yellow croaker, crucian carp, shrimp, and clam samples [36]. Furthermore, an ENR molecularly imprinted sensor using nitrogen-doped carbon dots (N-CDs)/MWCNTs as the electrode modification material and nicotinamide and L-cysteine as dual functional monomers also yielded detection results comparable to those of high-performance liquid chromatography (HPLC) in fish meat and lake water samples, further demonstrating the practical value of the MIP strategy in enhancing the selective recognition of fluoroquinolones in complex samples [80].

Immunosensing provides an alternative selective recognition strategy for the detection of fluoroquinolones. To address the issues of traditional immunosensors requiring additional signal markers and complex operating procedures, Huang et al. proposed a label-free competitive electrochemical immunosensor utilizing magnetic bead chemical and electrochemical conversion (C-ECC) for the detection of ENR. In this method, immunomagnetic beads (IMBs) are converted in situ into Prussian blue (PB) through acid dissolution and electrodeposition, allowing for both sample separation and signal generation without the need for additional labels. Moreover, the process of electrochemical refreshment (ER) effectively removes the insulating protein layer from the electrode surface, enhancing detection sensitivity. This method achieved a LOD of 4.17 pg/mL and its applicability was validated in fish and chicken samples [37]. To address the challenges of simultaneously detecting multiple fluoroquinolones and the cumbersome procedures involved in traditional labeled immunoassays, Chomthong et al. developed a label-free dual-channel electrochemical immunosensor on an origami-paper-based analytical device (oPAD) for the simultaneous detection of NOR and ENR (Figure 2c). The oPAD recognizes NOR and ENR through two antibody-immobilized zones, respectively, and utilizes two redox probes (thionine and ferrocenecarboxylic acid) to distinguish signals. The immunocomplex formed from antigen–antibody binding blocks electron transfer for the redox probes, thereby enabling label-free detection. The sensor yielded detection results comparable to those of HPLC and commercial test strips in milk, honey, and fish samples [38]. Furthermore, Lin et al. developed a high-affinity antibody through rational hapten design and constructed an electrochemical immunosensor for ENR detection, which was validated in water and fish samples [81].

Overall, electrochemical detection of fluoroquinolones primarily involves three strategies: direct electrocatalysis, molecular imprinting, and immunological recognition. Direct electrocatalytic detection utilizes the intrinsic electrochemical activity of molecules such as ENR, CIP, NOR, and GAT. It offers the advantages of simple operation, rapid response, and suitability for initial screening. However, it remains susceptible to interference from co-extracts and structural analogs in complex aquatic matrices. The molecular imprinting strategy enhances target recognition by constructing specific recognition sites, while the immunological recognition strategy relies on antigen–antibody specific binding and is suitable for highly selective detection and multiplex analysis. From a technological development perspective, this field has gradually shifted from single-signal amplification toward a combination of interfacial catalysis, selective recognition, and simultaneous detection of multiple targets. Future research could focus on the differential detection of fluoroquinolone structural analogs, maintaining selectivity in complex aquatic matrices, and the synergistic optimization of direct electrocatalytic and recognition-based detection strategies.

### 3.2. Sulfonamides

Sulfonamides are widely used in aquaculture and are a key focus of residue monitoring. Current detection research mainly targets the following antibiotics: sulfamethazine (SM2), sulfadimethoxine (SDM), SDZ, sulfamethoxazole (SMX), sulfathiazole (ST), and sulfanilamide (SN). Although these antibiotics possess certain inherent electrochemical responses, their signals are often weak and susceptible to interference from the surrounding matrix. As a result, electrochemical detection predominantly relies on combining highly active interfaces with specific recognition elements to improve analytical performance. Overall, the technical approach for detecting these antibiotics has developed into a dual strategy combining direct electrocatalysis with selective recognition. Methods based on molecular imprinting, aptamers, and immunological recognition constitute a significant portion of this research. Furthermore, detection methods are progressively shifting towards multi-target and portable platforms.

**Figure 3:**
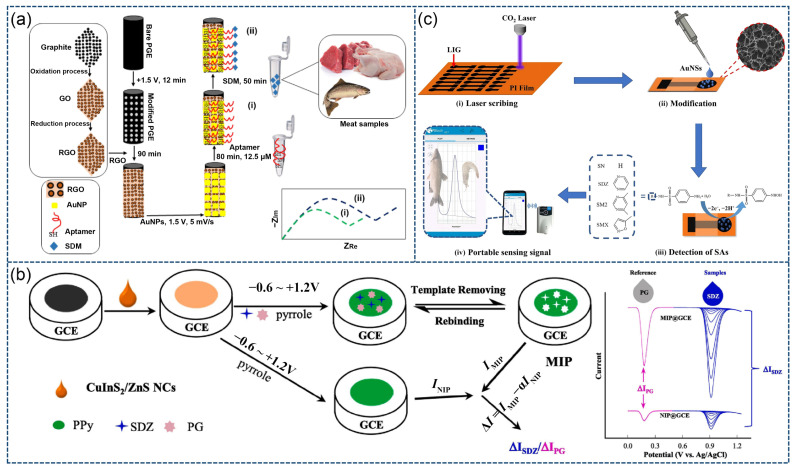
(**a**) Fabrication of AuNP/rGO/PGE-based aptasensor for SDM determination. Reproduced with permission from [40]. (**b**) Schematic diagram of the differential ratiometric MIP-based sensor for SDZ detection. Reproduced with permission from [41]. (**c**) Fabrication of AuNS/LIPG flexible electrode for multiplex detection of four sulfonamides. Reproduced with permission from [43].

Affinity-based recognition approaches include immunosensing and aptasensing. In the field of label-free immunosensing, in order to solve the problems of limited antibody loading and insufficient electron transfer efficiency of bare GCE, Liu et al. modified GCEs with COOH-MWCNTs-Fe_3_O_4_-GO nanocomposites for the detection of SM2. This composite combines the high conductivity and large specific surface area of COOH-MWCNTs, the electrochemical activity of Fe_3_O_4_, and the antibody immobilization capacity of GO, forming a layered and tubular interwoven interface that enhances antibody loading and signal response. The sensor demonstrated a rapid detection time of 30 min, a linear range of 0.01–100 ng/mL, a LOD as low as 0.003 ng/mL, and recovery rates of 94.4–109.0% in crayfish samples [39]. In the field of aptasensing, addressing the problem of traditional antibiotic detection relying on complex instruments and labeling steps, Mohammad-Razdari et al. developed a label-free impedimetric aptasensor based on a rGO/AuNPs-modified pencil graphite electrode for the detection of SDM (Figure 3a). In this system, rGO enhances the electrode’s conductivity and effective surface area, while AuNPs facilitate electron transfer and immobilize aptamers via Au–S bonds, thereby enabling highly sensitive recognition. The sensor achieved a linear range of 1.0 × 10^−15^–1.0 × 10^−5^ M, a LOD of 3.7 × 10^−16^ M, and recovery rates of 92–103% in fish, chicken, and beef samples, with results consistent with those obtained by HPLC [40].

Molecular imprinting sensing has become one of the most mature technologies for the detection of sulfonamides. To address the issues of insufficient sensitivity and limited selectivity in screen-printed carbon electrode (SPCE), Bai et al. constructed a molecularly imprinted electrochemical sensor on a SPCE by vertically growing covalent organic frameworks (v-COF) on carboxylated single-walled carbon nanotubes (SWCNTs-COOH) as a conductive support and integrating them with an SM2 MIP layer [33]. The vertical growth of the v-COF on SWCNTs significantly enhanced the electrical signal, resulting in an electrical signal 2.33 times stronger than that of a bare electrode. In addition, steam elution at 55 °C replaced traditional organic solvents, enhancing the environmental friendliness and convenience of the template removal process. The sensor achieved a LOD of 0.21 nM for SM2, with recovery rates varying from 95.0% to 104.8% in fish and milk samples. In another study, to address the significant nonspecific adsorption interference and sensitivity to fluctuations in preparation and detection conditions that affect reproducibility in traditional molecularly imprinted sensors, Yu et al. designed a differential ratiometric dual-mode molecularly imprinted electrochemical sensor for the detection of SDZ (Figure 3b). The design uses a CuInS2/ZnS nanocomposite material to enhance the signal. SDZ is used as the template molecule and propyl gallate (PG) is used as the reference molecule. The design utilizes the obvious separation of the oxidation peaks of SDZ and PG and uses ΔI_SDZ_/ΔI_PG_ as the quantitative signal. The differential mode eliminates interference from nonspecific adsorption, improving interference resistance by approximately one order of magnitude. The ratiometric mode, through self-correction with an internal reference, suppresses the influence of experimental condition fluctuations by a factor of 2.8–13.2. The sensor achieved a LOD of 2.1 nM for SDZ, with recovery rates of 96.2–105% in fish, beef, and milk samples [41]. Furthermore, a Cu-benzene-1,3,5-tricarboxylic acid metal–organic framework encapsulated with Fe and Ni single atoms (Cu-BTC@FeNi-SAs) combined with an MIP layer was utilized to detect SDZ, achieving recovery rates of 91.02–99.20% in seawater and fish samples [82]. This demonstrates that the combination of a single-atom catalytic interface with molecularly imprinted recognition helps improve the detection performance of sulfonamides in complex samples.

The key challenge in the direct electrocatalytic detection of sulfonamides lies in their inherently weak signal, which necessitates the development of highly active sensing interfaces. To address the problems of poor conductivity of traditional MOF materials and limited electrochemical performance of single MOF materials, Zhang et al. rapidly synthesized MIL-101(Fe)@N-Cu-MOF composites via ultrasonication methods and utilized them to modify GCEs for the detection of SMX. MIL-101(Fe) has a high specific surface area, while N-Cu-MOF has high conductivity. The synergistic effect of the two increased the electroactive surface area by more than five times compared with the bare GCE. The sensor exhibited a linear range of 0.01–150 µM, with a LOD as low as 0.0037 µM. Recovery rates for various samples, including carp, prawn, milk, serum, and urine, ranged from 82.7% to 118.3%, aligning with results of HPLC [42]. Furthermore, in response to the challenges of traditional electrodes in terms of flexibility, miniaturization and integration, Qiu et al. employed a one-step laser direct writing technique to fabricate gold nanoshell-decorated laser-induced porous graphene (AuNSs/LIPG) flexible electrodes on a polyimide substrate. These electrodes were integrated with a wireless Bluetooth portable workstation and a smartphone to construct a portable sensing platform (Figure 3c). The innovation of this platform lies in combining the advantages of flexible LIPG electrodes with the high electrocatalytic activity of AuNSs, enabling the rapid detection of four sulfonamides (SN, SDZ, SM2, and SMX). In fish and shrimp samples, the recovery rates ranged from 96.04% to 105.00%. This approach offers a low-cost, flexible, and portable solution for the rapid on-site screening of multiple sulfonamides in aquaculture [43]. In addition to these representative studies, the Sc-induced nickel–iron-layered double hydroxide (NiFeSc-LDH)/rGO-modified GCE facilitated the direct electrocatalytic detection of SM2, yielding recovery rates of 98.45–99.70% in beef, shrimp, milk, and honey samples [83]. A vanadate samarium-doped covalent organic framework, SmV/COF_TDBA-TTL_, constructed from terphenyl dicarboxylic acid and 2,4,6-tris(4-aminophenyl)-1,3,5-triazine monomers, was employed for the direct detection of SDZ [84]. A [(4,4′-bipy/P_2_Mo_17_Co)_6_] multilayer film was developed to modify a GCE for the direct detection of ST in large yellow croaker and South American white shrimp [85]. These studies demonstrate that interfacial materials such as LDHs, MOFs/COFs, and carbon-based composites can broaden the scope of direct detection of sulfonamides by improving electron transfer efficiency, increasing active sites, and enhancing electrocatalytic oxidation responses.

Overall, electrochemical detection of sulfonamides primarily involves four strategies: direct electrocatalysis, immunosensing, aptasensing, and molecularly imprinted sensing. Since the oxidation response of sulfonamides is typically weak, related research has focused on enhancing electron transfer and electrocatalytic responses by constructing highly active composite interfaces while incorporating recognition elements such as MIPs, antibodies, and aptamers to improve target recognition capabilities in complex samples. It is worth noting that the application of differential ratiometric signals, self-calibration strategies, and flexible, portable platforms has further driven the development of sulfonamide detection from single-target, high-sensitivity detection toward reliable analysis of complex samples and rapid on-site screening. Future research could focus on optimizing the selective differentiation of sulfonamide structural analogs, the stable amplification of weak oxidation signals, and signal correction strategies in complex aquatic matrices.

### 3.3. Amphenicols

Amphenicols, including CAP and florfenicol (FF), are crucial targets for monitoring antibiotic residues in aquatic animals. The nitro group in the CAP molecule is amenable to electrochemical reduction, making direct electrochemical methods the primary approach for detecting CAP [86]. In contrast, existing electrochemical detection methods for FF primarily depend on aptamer recognition and signal amplification strategies to effectively address the challenges of trace analysis in complex matrices.

**Figure 4:**
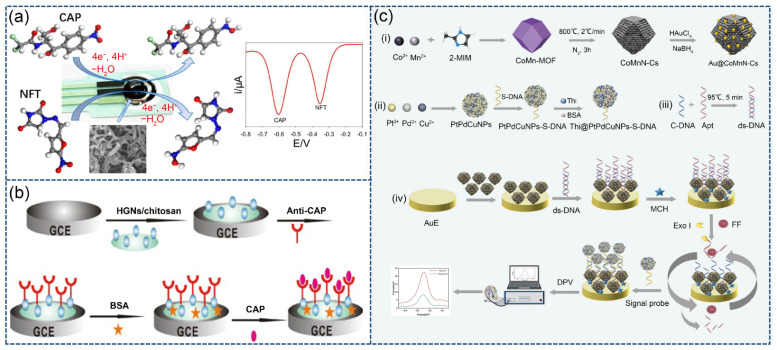
(**a**) Schematic diagram of the disposable magnetic FeCo@S,N-CNTs/SPE for electrochemical detection of CAP and NFT. Reproduced with permission from [46]. (**b**) Fabrication process of the HGNs/CS/GCE-based immunosensor. Reproduced with permission from [47]. (**c**) Schematic diagram of the preparation of Au@CoMnN-Cs, Thi@PtPdCu NPs-S-DNA, ds-DNA, and aptasensor for FF detection. Reproduced with permission from [49].

In the field of direct electrocatalysis, to address the problems of insufficient electrode interfacial activity and limited sensitivity in the electrochemical detection of CAP, Zhang et al. utilized the host–guest recognition properties of β-cyclodextrin (β-CD) to enrich CAP. By combining this with the electrocatalytic activity of CuO nanomaterials, they developed a β-CD/CuO/GCE for CAP detection. The synergistic interaction between β-CD and CuO enhanced electron transfer at the electrode interface, resulting in a linear detection range for CAP of 0.1–500 µM, a LOD of 0.05 µM, and a recovery rate of 96.0–102.4% in fish samples [44]. Another study addressed the need to simultaneously monitor CAP and metronidazole (MTZ) in aquatic animal samples due to their potential coexistence. Zhai et al. modified a GCE with silver nanoparticles and sulfonated functionalized graphene (AgNPs/SF-GR/GCE). By leveraging the synergistic enhancement of the electrochemical response resulting from the high conductivity of AgNPs and the large specific surface area of SF-GR, they achieved simultaneous detection of CAP and MTZ in shrimp samples using differential pulse stripping voltammetry (DPSV). In this method, the LOD for CAP was 0.01 µM, with spiked recovery rates ranging from 97.4% to 103.0% [45]. Furthermore, addressing the challenges of achieving rapid, controlled, and one-step fabrication with conventional modified electrodes, Qian et al. used a bimetallic MOF as a precursor to prepare an electrocatalyst consisting of FeCo alloy magnetic nanoparticles confined within S,N-co-doped carbon nanotubes (FeCo@S,N-CNTs), and modified a screen-printed electrode via a magnetically controlled method (Figure 4a). The innovation lies in the fact that the structural defects introduced by S,N co-doping, the synergistic catalytic effect of the FeCo alloy, and the confined carbon nanotube structure collectively enhance electrocatalytic activity and stability, while the strong magnetism of the FeCo alloy enables rapid and controlled assembly of the catalyst on the electrode surface. This sensor achieved simultaneous detection of CAP and furotamine (NFT), with a linear range of 0.05–320.0 µM and a LOD of 20 nM for CAP, and a linear range of 0.01–75.0 µM and a LOD of 3.5 nM for NFT. The sensor’s applicability was validated across various food matrices, including milk, honey, egg, fish, chicken, and pork [46]. In addition to the representative studies mentioned above, several interface systems have been utilized for the direct electrocatalytic reduction in CAP, including Bi_2_S_3_@GCN(graphitic carbon nitride)/SPCE, α-Fe_2_O_3_/SPE, Fe-Silk PNC/PGE (porous nitrogen-doped atomically dispersed Fe-Nx-C porous carbon nanosheet-modified pyrolytic graphite electrode), MoS_2_/SPE, and CoFe_2_O_4_@Fe-BTC/SPE [87,88,89,90,91]. These studies indicate that electrocatalytic interfaces such as metal sulfides, metal oxides, 2D materials, MOF-derived materials, and doped carbon materials can enhance the performance of direct electrochemical detection by improving electron transfer efficiency, increasing the number of active sites, and promoting the CAP reduction reaction.

In terms of selective recognition, to address the issues of low antibody loading and insufficient sensitivity in traditional immunosensors, Zhang et al. modified a GCE with a composite of hollow gold nanospheres (HGNs) and chitosan (CS) to develop a label-free electrochemical immunosensor for CAP detection (Figure 4b). The hollow structure and rough surface of the HGNs provide a large specific surface area and good biocompatibility, significantly increasing the antibody loading capacity. This resulted in a linear range of 0.1–1000 ng/mL a LOD as low as 0.06 ng/mL. Its applicability was validated in beef, fish, and pork, with detection results consistent with the HPLC method [47]. In the field of aptasensing, to address the issues of traditional aptasensors requiring the addition of redox probes and involving cumbersome operations, Kaewnu et al. employed Prussian blue (PB) as an embedded signal probe and utilized a chitosan-glutaraldehyde copolymer (CS-GA) to stabilize PB. They also incorporated AuNPs to enhance conductivity and increase the amount of immobilized aptamer, thereby constructing a CAP aptasensor based on a screen-printed electrode. The innovation lies in the use of PB as an embedded signal source, which eliminates the need for an external probe step. Meanwhile, electrode regeneration is achieved through constant-potential treatment at −0.6 V, enabling 24 cycles of reuse. The sensor exhibited a linear range of 1.0–6.0 × 10^6^ ng/L, a LOD of 0.65 ng/L, and recovery rates ranging from 88.0% to 100% in milk, shrimp pond water, and shrimp meat samples [48]. Furthermore, a research team developed two electrochemical aptasensors based on a competitive displacement strategy for detecting CAP in fish samples. The first sensor employed multi-metal ion-encoded nanospherical brushes as tracers, allowing for SWV detection following target-triggered tracer release and magnetic separation, achieving a LOD as low as 0.3 pg/mL [92]. The second sensor utilized CdS/PbS quantum dot-encoded dendritic nanoprobes, with the signal measured by SWV following acid-induced release of Cd^2+^ and Pb^2+^ from the quantum dots, resulting in a LOD of 0.33 pg/mL for CAP [93]. These studies demonstrate that combining aptamer recognition with metal ion encoding, quantum dot signaling, and magnetic separation strategies can further enhance their sensitivity to CAP for trace detection.

Furthermore, to address the issues of insufficient conductivity at the electrode interface and limited signal amplification efficiency in the detection of FF, Wang et al. modified a gold electrode with gold nanoparticles supported on MOF-derived Mn- and N-co-doped Co-C nanomaterials (Au@CoMnN-Cs). By combining Exonuclease I (Exo I)-assisted target recycling with the Thi@PtPdCuNPs-S-DNA signal probe, they constructed a dual-signal-amplification electrochemical aptasensor (Figure 4c). The porous structure and high conductivity of Au@CoMnN-Cs enhance the electrode interface performance and biomolecule loading capacity, achieving the first stage of signal amplification. Exo I cleaves the single-stranded aptamer to release FF and triggers a recycling reaction of the target, which allows a large amount of C-DNA to hybridize with the signal probe, thereby achieving the second stage of signal amplification. The sensor exhibited a linear range for FF of 1 × 10^−3^–1 × 10^3^ ng/mL, with a LOD as low as 5.28 × 10^−4^ ng/mL. The recovery rates in milk, egg, and shrimp samples were 98.0–100.3%, 96.3–100.9%, and 97.0–100.5%, respectively [49].

Overall, the electrochemical detection of CAP and FF exhibits distinct technical focal points. CAP exhibits relatively distinct electrochemical reduction activity. Therefore, related research has primarily focused on the construction of direct electrocatalytic interfaces and has gradually expanded to multi-target simultaneous detection and disposable electrode platforms. At the same time, immunological and aptasensing have further enhanced selectivity and trace-level detection capabilities in complex samples. FF detection, on the other hand, relies more heavily on aptamer recognition and cyclic amplification strategies to compensate for its insufficient direct electrochemical response. Future research may focus on improving the selective discrimination of the CAP reduction signal, the stability of FF aptamer recognition, and differentiated detection strategies for these two antibiotics in complex aquatic product matrices.

### 3.4. Tetracyclines

Tetracyclines, including tetracycline (TC), oxytetracycline (OTC), chlortetracycline (CTC), and doxycycline (DC), represent a class of broad-spectrum antibiotics that are extensively utilized in aquaculture [4]. These molecules exhibit typical amphiphilic properties and feature ionizable groups such as tricarbonylamide, phenolic diketone, and dimethylamine in their backbone. Consequently, their acid–base dissociation, molecular speciation, and interfacial behavior are significantly influenced by pH levels [94]. Furthermore, the presence of electroactive sites within the molecules, such as phenolic hydroxyl groups, tertiary amines, and hydroxyl groups, imparts intrinsic electrochemical activity to them [95]. Tetracyclines also readily form complexes with metal ions like Ca^2+^, Mg^2+^, and Cu^2+^, which further affects their solution behavior and electrochemical response characteristics [96]. However, their electrochemical processes are often accompanied by strong interfacial adsorption and surface contamination caused by oxidation products, which can easily lead to electrode passivation, signal attenuation, and reduced reproducibility [97]. Therefore, the challenge in detecting tetracyclines lies not only in obtaining a response signal but also in balancing sensitivity, selectivity, and stability in complex matrices, which necessitates the development of anti-fouling interfaces and the design of specialized functional materials [98].

**Figure 5:**
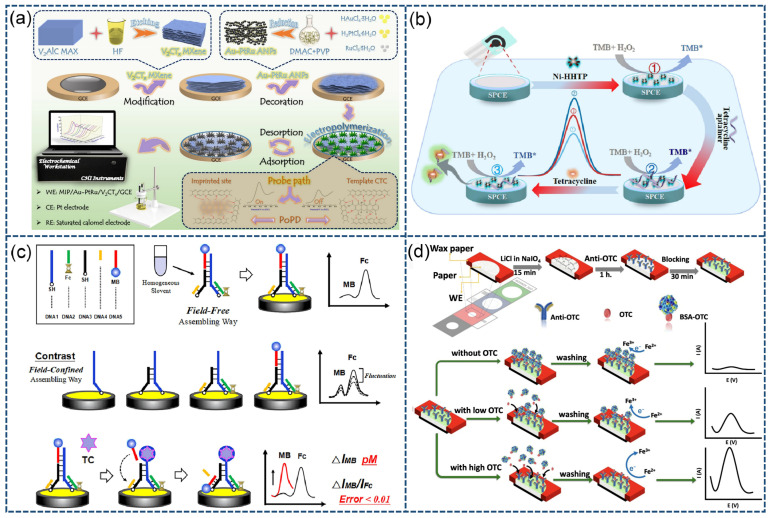
(**a**) Schematic preparation of Au-PtRu/V_2_CTx/GCE-based molecularly imprinted electrochemical sensor for CTC determination. Reproduced with permission from [50]. (**b**) Schematic illustration for preparation and working principle of the electrochemical aptasensor for TC detection based on aptamer-tuned nanozyme activity. Reproduced with permission from [52]. (**c**) Schematic of the ratiometric sensor based on inverted Y-type DNA for TC detection. Reproduced with permission from [54]. (**d**) Preparation and working principle of the disposable immunosensor for OTC detection. Reproduced with permission from [56].

In the detection of tetracyclines, molecularly imprinted sensors are often used to enhance the selectivity of target recognition. However, the poor conductivity of the MIP layer can easily limit the electrochemical response. To address this issue, Zhang et al. utilized V_2_CT_x_ MXene as a conductive support material and introduced an Au-PtRu nanoalloy to create a signal-enhancing interface. They then constructed a CTC MIP layer via a one-step electropolymerization process to develop a molecularly imprinted sensor (Figure 5a). This method utilizes the layered structure of V_2_CT_x_ MXene to provide loading sites and facilitate the dispersion of the Au-PtRu nanoalloy, while leveraging the high conductivity and synergistic catalytic effects of the Au-PtRu alloy to enhance interfacial electron transfer, thereby enabling the specific recognition and signal amplification of CTC. The sensor exhibited a linear range of 1–120 µM and a LOD of 0.16 µM. The recovery rates in fish, chicken, and pork samples ranged from 83.3% to 93.6%, and the results were consistent with those obtained using the HPLC method [50]. Devkota et al. developed a molecularly imprinted sensor on a low-cost SPCE for the sensitive detection of TC. This electrode was modified with molecularly imprinted overoxidized polypyrrole (MIOPPy) and AuNPs, where MIOPPy provides specific recognition sites, AuNPs compensate for the reduced conductivity of overoxidized polypyrrole, and sodium dodecyl sulfate (SDS) further enhances the response by promoting the enrichment of TC on the electrode surface. The sensor reached a LOD of 0.65 µmol/L and a linear range of 1–20 µmol/L. Its applicability was validated in shrimp samples, yielding results comparable to those obtained by LC-MS [51].

In recent years, the detection of tetracyclines has increasingly focused on aptasensing methods. To address the issue that traditional MOF nanoenzyme aptasensors typically require aptamer functionalization and complex coupling reactions, Wang’s team utilized the peroxidase activity of a conductive MOF of Ni^2+^-2,3,6,7,10,11-hexahydroxytriphenylene (Ni-HHTP) to develop an electrochemical aptasensor for detecting TC (Figure 5b). This method enables noncovalent adsorption of the aptamer via π–π stacking and utilizes TC-induced conformational changes in the aptamer to modulate the catalytic activity of Ni-HHTP toward the TMB/H_2_O_2_ system, thereby generating a signal. The sensor achieved a low LOD of 1.9 pM and was successfully applied to the detection of TC in grass carp and milk samples [52]. Subsequently, addressing the issue of complex fabrication caused by the separation of the aptamer-immobilized matrix from the signal output unit, the same team used a composite of polyaniline@copper-1,3,5-benzenetricarboxylic acid (PAN@Cu-BTC) as a bifunctional interface. This composite both immobilized the aptamer through coordination between Cu^2+^ and the 5′-phosphate group and served as an endogenous electrochemical signal source. The sensor achieved TC detection through a target-induced decrease in the electrochemical signal, with a LOD as low as 0.32 pM. Recovery rates in grass carp meat ranged from 90.0% to 102.0% [53]. In terms of DNA configuration programming, Wang et al. addressed the issue that conventional surface-confined DNA sensors are susceptible to steric hindrance and nonspecific signal interference by designing a ratiometric sensor based on an inverted Y-type DNA conformation for TC determination (Figure 5c). They amplified the methylene blue (MB) response through a toe-hold strand displacement strategy and used a built-in ferrocene (Fc) reference, achieving a LOD of 28.4 pM and recovery rates ranging from 98.0% to 103.0% in fish samples [54]. In terms of signal amplification, Pan et al. developed a “signal-on” type OTC aptasensor based on a PdSn/MIL101(Fe) nanoenzyme and a signal probe. The sensor utilizes the high conductivity and peroxidase-like activity of PdSn/MIL101(Fe) to amplify the TMB/H_2_O_2_ electrochemical signal, achieving a linear range of 100 fg/mL–100 ng/mL and a LOD as low as 8.13 fg/mL. Its applicability was validated in fishmeal quality control samples [55]. In addition, Ce-MOF-808@CeO_2_-modified SPE was also used to construct a TC aptasensor. This method utilizes nanoenzyme activity to generate a signal and has been validated using shrimp samples, further demonstrating that the combination of MOF-derived nanoenzyme interfaces and aptamer recognition can enhance the detection of trace amounts of tetracyclines [99].

To address the issues that traditional immunosensors rely on labeled probes and complex modification steps, Jampasa et al. developed a paper-based competitive immunosensor for the detection of OTC (Figure 5d). This design employs filter paper as the substrate, onto which anti-OTC antibodies are immobilized via chemical functionalization. The detection principle is based on the competitive binding between OTC and OTC-BSA, with [Fe(CN)_6_]^3−/4−^ serving as the redox probe for signal readout. It employs a “signal-on” label-free mode, in which the presence of the target OTC reduces the binding of OTC-BSA at the antibody interface, thereby decreasing the interfacial blocking effect and enhancing the current response of the redox probe. The sensor achieved a LOD of 0.33 ng/mL, with a linear range of 1–200 ng/mL. Additionally, its practicality was validated in milk, honey, and shrimp samples [56].

In the field of direct electrocatalytic detection, although tetracyclines possess inherent electrochemical activity, issues such as interfacial adsorption and electrode fouling are particularly prominent. Therefore, related research has primarily focused on modifying functional materials to enhance electron transfer, improve electrode stability, and amplify the response signal. Previous studies have employed a GCE modified with mixed-valent ruthenium oxide-ruthenium cyanide (mvRuO-RuCN) and a nickel-implanted boron-doped diamond thin film electrode (Ni-DIA) for the direct electrochemical detection of four tetracyclines (TC, OTC, CTC, and DC), demonstrating their applicability in shrimp samples [100,101]. A screen-printed gold electrode (SPGE) was also utilized for the direct detection of TC, OTC, and CTC, and its efficacy was validated in chicken and shrimp samples [102]. Subsequently, the Gan research group modified a GCE with zinc cation-exchanged montmorillonite (Zn-Mt) and iron/zinc cation-exchanged montmorillonite (Fe/Zn-MMT) for the direct electrochemical oxidation of OTC and TC, respectively, validating their results in fish, shrimp, and chicken samples [103,104]. In recent years, innovative systems such as WO_3_/rGO/GCE, silver-doped zinc ferrite nanoparticle-embedded chitosan-functionalized carbon nanofiber-modified SPCE (AgZFO/CHIT-CNF/SPCE), and fluorine-doped activated carbon combined with deep eutectic solvent-modified SPE (DES-F-AC/SPE) have broadened the applications of direct electrocatalytic methods for the detection of TC and OTC in aquatic products [105,106,107]. These studies have further expanded the range of electrode materials and practical sample applications for the direct electrochemical detection of tetracyclines.

Overall, electrochemical detection of tetracyclines primarily involves strategies such as direct electrocatalysis, molecular imprinted sensing, aptasensing, and immunosensing. Since these molecules possess inherent electrochemical activity but are susceptible to factors such as pH, metal complexation, interfacial adsorption, and contamination by oxidation products, related research must not only enhance the electrochemical response but also improve detection stability and adaptability to complex matrices. In recent years, advancements in nanoenzyme signal amplification, DNA conformation regulation, ratiometric signal correction, and the development of portable paper-based platforms have driven the evolution of tetracycline detection from a simple focus on sensitivity enhancement toward reliable analysis of complex samples and rapid on-site screening. Future research could focus on optimizing anti-contamination interfaces, sample pretreatment, and signal correction strategies to improve detection stability and result reliability in complex aquatic samples.

### 3.5. Nitrofurans

Nitrofurans, including furazolidone (FZD), furaltadone (FLD), nitrofurazone (NFZ), and NFT, were previously prevalent in anti-infective therapy for aquaculture and other food-producing animals [108]. However, due to the genotoxic and carcinogenic risks associated with these antibiotics and their metabolites, their use has been banned or is no longer authorized in food-producing animals within the European Union and other countries. Structurally, nitrofuran molecules typically feature a 5-nitrofuran moiety, and the nitro group is readily reducible at the electrode interface, facilitating the detection of signals [109]. Leveraging this property, direct electrochemical detection has long been an important research approach for analyzing this class of antibiotics. In recent years, advancements such as molecularly imprinted recognition, arrayed electrode platforms, and portable electrochemical sensors have been continuously developed to improve selectivity, enhance anti-interference capabilities, and allow for on-site applications in complex matrices [61,110,111].

To address the issues of weak electrode reaction signals and insufficient selectivity in the direct detection of nitrofurans, Amalraj et al. synthesized a ZnO–ZnCo_2_O_4_ nano-heterostructure via a one-step hydrothermal-calcination method and used it to modify a GCE for the detection of FLD. The innovation of this method lies in utilizing the ZnO–ZnCo_2_O_4_ heterojunction to increase the electroactive surface area, provide more active sites, and promote electron transfer, thereby enhancing the electrochemical reduction response of FLD. The sensor showed a linear range of 0.01–4.68 µM and 15–100 µM, with a LOD as low as 1.46 nM, and its results were validated against HPLC data using fish tissue samples [57]. Subsequently, addressing the challenge that the detection of a single nitrofuran could not meet the requirements for simultaneous monitoring, Adane et al. constructed a composite interface consisting of Au-Ag alloy nanocoral clusters (Au-Ag-ANCCs), ZnO nanoparticles (ZnO-NPs), and polyethylene oxide (PEO) for the simultaneous detection of NFT and FZD. This design leverages the high conductivity and catalytic activity of Au-Ag-ANCCs, the electron transfer-promoting ability of ZnO-NPs, and the interface stabilization and mass transfer enhancement provided by PEO. The synergistic interaction of these three components significantly reduces charge transfer impedance and increases the active surface area. The sensor achieved detection limits as low as 0.26 pM and 0.023 pM for NFT and FZD, respectively. Its applicability was validated with various samples, including fish, chicken, honey, milk, and wastewater, achieving recovery rates ranging from 96.3% to 102.4% for NFT and 96.4% to 102.8% for FZD [58]. To address the issues of low integration and difficulty in rapidly screening multiple nitrofurans associated with traditional electrodes, Sun et al. employed a LIGE array as a low-cost integrated platform and sequentially modified it with N-doped carbon-stabilized atomically dispersed Cu sites (CuNC) derived from Cu@ZIF-8 and S-doped g-C_3_N_4_ ultrathin nanosheet-supported Ru (Ru@S-CN). This design leverages the porous conductive structure of LIGE, the Cu single-atom sites, and the catalytic activity of Ru@S-CN to synergistically enhance electron transfer and target adsorption capabilities, enabling the rapid detection of NFT, FZD, NFZ and FLD. This CuNC/Ru@S-CN/LIGE-based platform achieved low LODs (NFT: 0.98 nM, FZD: 0.25 nM, NFZ: 1.6 nM, FLD: 8.7 nM) and demonstrated applicability in aquaculture water, sediment, grass carp, and *Penaeus orientalis*, with recoveries ranging from 90.87% to 106.4% [59]. In addition, several systems have also been utilized for the direct electrochemical detection of nitrofurans. These include ZnO/ZnFe_2_O_4_/SPE, Co_3_S_4_@MoS_2_/GCE, PrVO_4_/GCE, GdVO_4_/SPCE, hollow MIL-101/GCE, TiO_2_@rGO/GCE, electrochemically functionalized graphene nanosheets (EGS)/GCE, and [Ru-PMo_12_/PDDA-GO]_3_/GCE. Their effectiveness has been validated in aquatic product samples such as shrimp, tuna, river fish, and crayfish [112,113,114,115,116,117,118,119]. This indicates that metal oxides, rare-earth vanadates, graphene-based materials, and MOFs are also good choices for the interface design.

**Figure 6:**
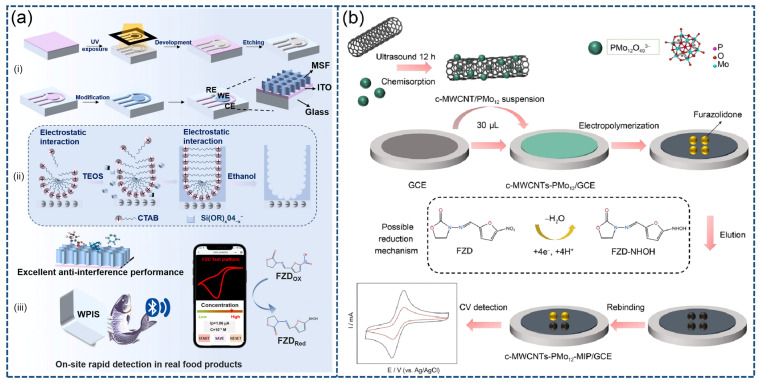
(**a**) Schematic of the preparation process of MSFM and the formation process of MSF coating on the ITO, and their integration into a wireless portable intelligent sensor for on-site rapid detection of FZD. Reproduced with permission from [61]. (**b**) Schematic diagram of the preparation of a molecularly imprinted sensor for FZD detection. Reproduced with permission from [62].

Portable on-site detection is one of the key development directions for the electrochemical analysis of nitrofurans. To address the issues of operational inconvenience, complex modification steps, and limited on-site applicability associated with traditional three-electrode systems, Jiang et al. prepared an integrated three-electrode laser-induced graphene (LIG) array for the detection of NFZ by laser engraving commercial polyimide tape. Their innovation lies in modulating the 3D porous structure of LIG through laser power control. The engraved graphene array at 50% laser power percentage (LIG-50) array exhibits high conductivity, low charge transfer resistance, and good stability, enhancing the NFZ oxidation response without the need for complex modifications. The sensor achieved a linear range of 0.2–8 µM, a LOD of 0.035 µM, and was successfully applied to the direct detection of NFZ in fish samples [60]. To address the issues of bulky traditional equipment, the susceptibility of complex fish samples to electrode contamination, and the typical need for sample pretreatment, Yan et al. developed a wireless portable intelligent sensor (WPIS) for the on-site detection of FZD in fish samples (Figure 6a). This platform integrates a mesoporous silicon film microelectrode (MSFM) with a Bluetooth-enabled smartphone readout system. It utilizes size exclusion, electrostatic repulsion, and nanoscale confinement effects to reduce matrix interference from proteins, lipids, and other components, enabling real-time detection of FZD in fish samples without pretreatment. The sensor demonstrated a detection range of 0.1–100.0 µmol/L and a LOD of 0.05034 µmol/L, demonstrating good resistance to contamination, repeatability, and potential for on-site applications [61].

Although nitrofurans possess strong intrinsic electrochemical activity, molecularly imprinted sensors can further enhance detection selectivity in complex samples through specific recognition sites. To address the issue of insufficient selectivity in direct electrocatalytic detection among structural analogs and complex matrices, Huang et al. fabricated a molecularly imprinted sensor for the detection of FZD by combining carboxylated multi-walled carbon nanotubes (c-MWCNT) with phosphomolybdic acid (PMo_12_), followed by the electropolymerization of a MIP film (Figure 6b). In this design, the high conductivity of c-MWCNT facilitated electron transfer, the multi-electron redox properties of PMo_12_ enhanced the electrocatalytic response, and the imprinted cavities ensured specific recognition. This sensor acquired a LOD of 3.38 nM and was successfully applied to the determination of FZD in shrimp samples, yielding satisfactory recovery results [62]. This study demonstrates that, for highly electroactive nitrofurans, the primary value of the MIP strategy lies not in replacing direct electrocatalysis, but in enhancing selectivity and resistance to interference in complex matrices while maintaining signal response.

Overall, the electrochemical detection of nitrofurans primarily relies on direct electrocatalytic reduction, which is closely related to the inherent electrochemical activity conferred by their 5-nitrofuran structure. Compared to certain antibiotics with weaker electrochemical responses, nitrofurans are better suited for rapid detection via highly active electrode interfaces, and can be further developed into multi-target arrays and portable on-site detection platforms. At the same time, because molecules such as FZD, NFT, NFZ, and FLD have similar structures, and coexisting components in aquatic product samples (such as proteins, lipids, and pigments) can easily cause interference, relying solely on direct electrochemical responses may still result in insufficient selectivity. Therefore, the key to detecting these drugs lies in combining their inherent electrochemical activity with molecularly imprinted recognition, anti-contamination interfaces, and integrated readout platforms to achieve rapid and reliable screening of banned drugs in complex aquatic samples.

### 3.6. Others (Macrolides, Aminoglycosides, and β-Lactams)

In addition to the five classes of antibiotics previously mentioned, studies on electrochemical detection in aquatic animals also encompass macrolides, aminoglycosides, and β-lactams. Macrolides demonstrate specific electrochemical responses, which are closely linked to their amine functional groups and protonation states [120]. Due to their highly polar structures, aminoglycosides are often detected using selective recognition methods such as aptasensing [121,122]. The electrochemical behavior of β-lactams displays significant dependence on their structure, influenced by both the core structure and the side chains [123]. In complex matrices, the incorporation of recognition elements, such as antibodies, is frequently employed to enhance selectivity [124].

Currently, research on electrochemical sensing of macrolides in aquatic products remains relatively limited. To address the challenge of simultaneously achieving conductivity, electrocatalytic activity, and the ability to detect multiple components at a single-material interface, Adane et al. modified a GCE with a nanocomposite composed of thermally annealed gold–silver alloy nanoporous matrices (TA-Au-Ag-ANpM), iron-doped polyaniline (Fe-dop-PANI), and nickel oxide nanoparticles (NiO-NPs) for the simultaneous detection of azithromycin (AZM) and ENR. This design leveraged the high conductivity and electrocatalytic properties of the Au-Ag alloy, the conductive polymer interface and potential binding sites of Fe-dop-PANI, and the electron-mediating role of NiO-NPs, resulting in an approximately fourfold increase in the electroactive area compared to a bare electrode and a significant reduction in charge transfer resistance. This sensor achieved effective peak separation and simultaneous quantification of AZM and ENR, with detection limits as low as 0.053 pM and 0.013 pM, respectively. Its applicability was confirmed in chicken meat, eggs, fish, river water, and lake water samples, yielding recovery rates between 96.4% and 102.8% [63].

**Figure 7:**
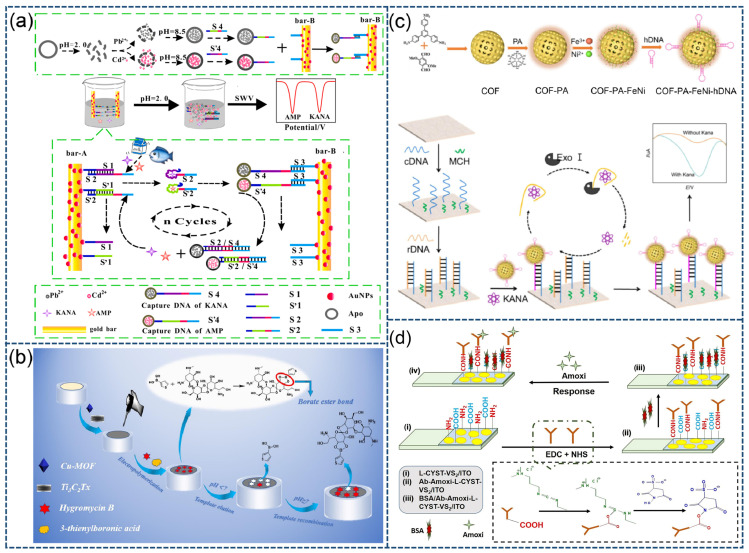
(**a**) Schematic of the preparation of encoded signal tags and illustration of the simultaneous detection of KANA and AMP based on a double stirring bar-assisted target recycling strategy. Reproduced with permission from [65]. (**b**) Schematic illustration of the preparation of MIP/Cu-MOF/Ti_3_C_2_T_x_/AuE and its electrochemical detection of hygromycin B. Reproduced with permission from [66]. (**c**) Schematic of the preparation procedure of the COF-PA-FeNi-hDNA probe and illustration of the principle of the electrochemical sensor for KANA detection based on Exo I and COF-PA-FeNi nanomaterial. Reproduced with permission from [68]. (**d**) Schematic of the preparation of the L-CYST-VS_2_/ITO electrode and its mechanism for detecting Amoxi. Reproduced with permission from [69].

Aminoglycosides primarily include kanamycin (KANA), tobramycin (TOB), and hygromycin B. Because their molecular structures contain multiple amino and hydroxyl groups, direct electrochemical responses are typically weak. Therefore, their detection predominantly utilizing the aptasensing approach. To address the challenges of signal discrimination and insufficient sensitivity for trace-level detection in the simultaneous detection of multiple antibiotic residues, Gan’s team developed an electrochemical aptasensor utilizing endonuclease- and exonuclease-assisted dual recycling amplification for the simultaneous detection of KANA and CAP. This method utilized hierarchical porous UiO-66-NH_2_ loaded with MB and Fc as distinguishable signal labels, alongside gold nanoparticle-modified stirring bars for target recognition, separation and cyclic amplification. This method achieved detection limits of 35 fM and 21 fM for KANA and CAP, respectively, and was validated in milk and fish samples [64]. In the following year, the team further proposed an enzyme-free, double stirring bar-assisted target recycling strategy, utilizing Cd^2+^- and Pb^2+^-loaded apoferritins as metal-ion-encoded probes to enable the simultaneous detection of KANA and ampicillin (AMP) (Figure 7a). This strategy avoided the cost and stability issues associated with enzyme-based methods while reduced matrix interference through stirring bar separation, achieving detection limits of 18 fM and 15 fM for KANA and AMP, respectively, and was validated in milk and fish samples [65]. In addition to aptasensing, molecularly imprinted sensing offers a complementary strategy for the selective detection of aminoglycosides. To address the issues of non-uniform recognition sites in traditional non-covalent MIPs and insufficient conductivity of MOFs, Wang et al. utilized the high specific surface area of Cu-MOFs and the excellent conductivity of Ti_3_C_2_T_x_ to construct a composite interface and prepared a molecularly imprinted sensor for detecting hygromycin B by electropolymerizing the MIP on a gold electrode (Figure 7b). This sensor utilized reversible borate ester bonds to enable the controlled elution and rebinding of the template molecule, achieving a LOD of 1.92 nM. Its applicability was validated in fish, pork, and chicken samples, with results consistent with those obtained by HPLC-MS/MS [66].

In recent years, electrochemical detection of aminoglycosides has gradually evolved toward engineered integration and flexible platforms. In response to the limitations of traditional single-material signal amplification capabilities and the insufficient interface conductivity in TOB trace detection, Deng et al. developed a dual-engine electrochemical aptasensor. This design employs AuNWs-Tb/Sn-MOF-on-Ce-MOF as the signal label, where the Sn-MOF-on-Ce-MOF heterostructure enhances the loading of toluidine blue (Tb) and signal response, while the AuNWs increase the binding sites for signal probes. Concurrently, acetylene black/polyethyleneimine/gold nanoparticles (AB/PEI-AuNPs) serve as the conductive substrate, increasing the effective electrode area and electron transfer efficiency. The synergistic interaction of these two modules enabled the sensor to achieve a low LOD of 0.19 fg/mL, and its applicability was validated in fish, chicken, duck, milk, and honey samples [67]. Meanwhile, to address the issues of insufficient flexibility in traditional rigid electrodes and the limited conductivity and catalytic activity of COF materials, Hou et al. integrated a flexible carbon cloth electrode, a phytic acid-regulated bimetallic covalent organic framework (COF-PA-FeNi), and an Exo I-assisted DNA cycling technique for the detection of KANA (Figure 7c). In this design, phytic acid not only introduces phosphorus atoms into the COF but also provides coordination sites for Fe^3+^ and Ni^2+^, resulting in a highly efficient bimetallic catalytic interface. Exo I-assisted target cycling further converts low-abundance KANA recognition events into abundant DNA signals, thereby achieving signal amplification. The sensor achieved a LOD of 64.7 fM and demonstrated excellent detection reliability and resistance to interference in fish, milk, and honey samples [68]. In addition, previous studies have reported aptasensors for the single-target detection of KANA and the dual-target detection of KANA and CAP, which have been validated in fish samples [125,126]. Another study utilized microplatinum electrodes and dendritic gold deposition technology to develop a microaptasensor, enabling real-time monitoring of the passive uptake of KANA in a single salmon egg [127].

Research on electrochemical detection of β-lactams is relatively limited, with immunosensing of amoxicillin (Amoxi) serving as the primary example. To address the issues of Amoxi’s weak electrochemical response and the difficulty of selective detection in complex samples, Kumar et al. modified an ITO electrode with L-cysteine-capped VS_2_ quantum dots (L-CYST-VS_2_QDs) to construct a label-free electrochemical immunosensor for the detection of Amoxi (Figure 7d). The L-CYST enhances the aqueous dispersibility and film-forming stability of VS_2_QDs, while also providing a stable interface for antibody immobilization. This sensor achieved a linear range of 1 pM–100 µM, a LOD of 0.5 pM, and its practical applicability was validated in fish samples [69]. The same team also developed an electrochemical immunosensor using a composite of chitosan (CS) and thioglycolic acid-capped VS_2_ quantum dots for the detection of Amoxi in fish samples [128]. These indicate that the electrochemical detection of β-lactams remains largely in the early stages of antibody recognition and quantum dot-enhanced sensing.

Compared with the aforementioned classes of antibiotics, there has been relatively little research on the electrochemical detection of macrolides, aminoglycosides, and β-lactams in aquatic products. However, the detection strategies for these three classes differ significantly. For macrolides, direct electrocatalytic detection is currently the primary approach, utilizing multicomponent composite interfaces to enhance the electrocatalytic response and enable simultaneous analysis of multiple targets. Due to their weak direct electrochemical responses, aminoglycosides rely more heavily on aptamer recognition, molecularly imprinted recognition, and signal amplification strategies. Among these, advancements in metal ion encoding, enzyme-assisted cycling, target recovery, and flexible electrode platforms have enhanced their trace detection capabilities. Reports on the electrochemical detection of β-lactams remain relatively limited, currently relying primarily on antibody recognition and quantum dot-enhanced interfaces. Overall, research on these three classes of antibiotics focuses not on simply enhancing direct electrochemical responses, but on selecting appropriate recognition elements, signal amplification methods, and interface materials based on the structural characteristics of the targets to improve detection sensitivity and selectivity in complex aquatic samples.

## 4. Sample Pretreatment for Electrochemical Detection of Antibiotics in Aquatic Animals

### 4.1. Necessity of Sample Pretreatment and Its Distinction from Traditional Methods

Aquatic animal tissues are abundant in proteins, lipids, inorganic salts, and endogenous small molecules, making them a complex food matrix. Antibiotic residues usually exist at trace levels and may either bind to or be adsorbed by the tissue components. To facilitate the electrochemical analysis of actual aquatic samples, pretreatment is essential to produce a sample solution that is suitable for detection. This process is crucial for effectively releasing target analytes from the tissue, minimizing interference from co-extracted matrix components, and ensuring compatibility between the sample and the analytical system [129,130].

Traditional LC-MS/MS and HPLC methods typically follow systematic steps that include extraction, purification, concentration, redissolution, and quantitative calibration. These processes aim to enhance recovery, minimize matrix effects, and ensure accurate quantification [131]. In contrast, matrix effects in electrochemical detection are primarily concentrated at the sensing interface. Since electrochemical sensors come into direct contact with the sample extract, co-extracted components such as proteins, lipids, and cell debris can adsorb or deposit onto the electrode surface. This can obstruct active sites, impede electron transfer, and adversely impact the binding capacity of the antibody, aptamer, or MIP layer. Research on anti-fouling interfaces suggests that nonspecific adsorption and interfacial contamination in complex samples are significant factors that limit the sensitivity, reproducibility, long-term stability, and reliability of electrochemical sensors for real-sample detection [132,133].

Therefore, the electrochemical detection of antibiotics in aquatic animal samples still necessitates sample pretreatment, although this process can be somewhat simplified compared to traditional methods. Traditional techniques prioritize comprehensive purification and high-accuracy confirmation, while pretreatment for electrochemical detection focuses more on compatibility with the sensing interface. In addition to maintaining advantages such as speed, low cost, and portability, pretreatment should facilitate efficient target release, the removal of major contaminants, adjustments to pH and solvent conditions, and signal stabilization. Thus, the core of pretreatment in electrochemical detection lies in achieving a balance between target extraction efficiency, interfacial antifouling capability, and electrochemical signal stability.

### 4.2. Sample Pretreatment for Electrochemical Detection of Antibiotics in Aquatic Animals

This section summarizes the existing sample pretreatment procedures for the electrochemical detection of antibiotics in aquatic animal samples. Common steps typically involve: sample homogenization or grinding, extraction using organic solvents or buffers, centrifugation or filtration, and, when necessary, defatting or purification. After these steps, samples may be concentrated or diluted and then resuspended in PBS, acetate buffer, or other supporting electrolytes before electrochemical detection. Although there are similarities in the pretreatment methods for different classes of antibiotics, the specific approach used can vary based on the solubility, pH properties, and chelating capacity of the target antibiotic, as well as the composition of the sample matrix. Table 2 provides an overview of common pretreatment strategies for the major classes of antibiotics in electrochemical detection in aquatic animal samples.

As indicated in Table 2, there is currently no standardized protocol for the pretreatment of aquatic animal samples before the electrochemical detection of antibiotics. Nevertheless, most methods primarily involve extraction, centrifugation and filtration, along with necessary concentration and redissolution steps, as well as adjustments to pH or electrolyte conditions prior to detection. Fluoroquinolones are typically extracted using organic solvents or acidic systems, with common examples including ethanol/acetic acid, ethyl acetate/ammonia, and hydrochloric acid. In contrast, sulfonamides are predominantly extracted using acetonitrile, although some studies also utilize systems such as ethyl acetate, acetonitrile/PBS, or PBS. The post-extraction processing for these two classes of antibiotics is relatively simplified, typically involving centrifugation, filtration, concentration and redissolution, or pH adjustment [32,33,34,41].

In contrast, the pretreatment process for amphenicols and tetracyclines is more reliant on the structural characteristics of the targets as well as the nature of matrix interferences. For amphenicols in certain fish and shrimp samples, it is necessary to employ a combination of dehydration using anhydrous sodium sulfate, defatting with n-hexane, and redissolution in PBS to address issues related to lipids and hydrophobic co-extractants [44,93]. When it comes to tetracyclines, greater emphasis must be placed on metal chelation and pH sensitivity. As a result, extraction is frequently conducted using an EDTA-McIlvaine buffer or an EDTA/acidic buffer/acetonitrile mixture to improve target release efficiency and reduce the effects of variations in chelation states on detection [101,102,103].

The extraction methods for nitrofurans are quite varied, including the use of acetonitrile/water, acetonitrile, trichloroacetic acid, and deep eutectic solvents. These techniques typically involve several steps, such as sonication, centrifugation, filtration, nitrogen drying for concentration, and redissolution in a buffer. These processes aim to enhance the extraction efficiency of the targets and improve the solution conditions prior to detection [60,116,119].

Given the limited electrochemical studies on macrolides, aminoglycosides, and β-lactams in aquatic animal samples, as well as the absence of comprehensive information regarding real-sample pretreatment, this section does not categorize their pretreatment strategies. In comparison to HPLC or LC-MS/MS, electrochemical detection often minimizes the need for complex purification and labor-intensive separation steps. Nevertheless, suitable pretreatment remains essential to ensure the effective release of targets, maintain electrode stability, and achieve reproducibility in results.

It should be noted that most current electrochemical sensing studies still primarily rely on spiked recovery experiments to validate method feasibility, and pretreatment protocols are often empirically optimized for a single target and a single matrix, with a lack of unified evaluation criteria. Although some studies have reported low detection limits and high recovery rates, there remains insufficient discussion of matrix effects, electrode contamination, deactivation of the recognition layer, and variations among different sample batches. Therefore, future optimization of sample preparation methods should not only focus on the extraction efficiency of the target antibiotics but also place greater emphasis on the compatibility of the extraction solution with the electrochemical sensing interface. It is essential to systematically evaluate the effects of solvent residues, pH, salinity, and co-extracted proteins and lipids on electron transfer, probe diffusion, and the stability of the recognition element in order to improve the reliability and reproducibility of real-sample detection.

## 5. Future Perspectives

Future research on electrochemical (bio)sensors for antibiotic residues in aquatic products should shift from simply pursuing lower detection limits to systematically improving method reliability, platform reproducibility, and the transferability of results to field applications. Before proposing specific optimization strategies, it is necessary to compare the practical application potential and limitations of large-scale implementation of different sensor platforms in the detection of antibiotic residues in aquatic products, taking into account their respective characteristics.

Considering the application characteristics of the four sensor types, direct electrochemical sensors show significant practical potential for the rapid screening of antibiotic residues in aquatic products. These sensors are simple to operate, quick to respond, and relatively low-cost, making them particularly suitable for on-site detection when combined with SPE, paper-based electrodes, LIGE, and portable workstations. However, they remain limited by factors such as interference from complex matrices, peak overlap, electrode contamination, and insufficient selectivity. Aptasensors also hold significant potential for highly selective, highly sensitive detection and multi-target analysis. However, their application remains constrained by factors such as the screening of high-affinity aptamers, conformational stability in complex matrices, and consistency in interface preparation. In contrast, molecularly imprinted sensors offer advantages such as a stable recognition layer and lower preparation costs. However, their use in antibiotic detection in aquatic products is currently limited to laboratory validation, and issues such as the uniformity of imprinted sites, template removal, cross-reactivity, and inter-batch reproducibility need to be further addressed. Although immunosensors exhibit high specificity, the cost of antibodies, storage stability, and inter-batch variations limit their large-scale on-site application. Overall, the ability to withstand interference from complex matrices, consistency in batch production of electrodes, stability of the recognition interface, standardized sample pretreatment, and portable detection procedures remain the primary factors affecting the large-scale application of these sensor platforms. Furthermore, insufficient validation with real samples and comparisons with confirmatory methods can also impact the reliability of these methods and their regulatory acceptance.

In addition to the type of platform itself, the stability, reusability, and reproducibility of modified electrodes are also important factors affecting their practical application. Based on the published studies, the reliability of modified electrodes is typically evaluated through storage stability, repeatability of measurements, and reproducibility across electrodes. Storage stability is typically assessed at 4 °C or room temperature, with evaluation periods generally ranging from several days to several weeks. A few studies extend this to several months. Overall, most modified electrodes retain a high percentage of their initial response during the corresponding storage periods. However, storage temperature, humidity, sealing methods, and storage media vary across studies, making it difficult to directly compare stability among different sensors. Regarding reusability, some MIP-based sensors, direct electrochemical sensors, and a few immunosensors/aptasensors can be reused multiple times through continuous measurements, elution, or regeneration cycles. For example, certain ENR, SDZ, CAP, CTC, and OTC sensors maintain relatively stable responses after multiple measurements or regeneration cycles [32,41,47,48,50,82,103]. However, for most immunosensors/aptasensors, factors such as target binding, contamination by complex matrices, or desorption of the recognition layer may still limit their long-term reusability. For disposable platforms such as SPE, paper-based electrodes, LIGE, and flexible electrodes, reusability is generally not a primary objective. Their advantages lie in lower cost, avoidance of cross-contamination, and ease of rapid on-site screening.

Regarding the reproducibility of electrode preparation, existing studies typically use a small number of independently prepared electrodes for parallel testing and evaluate inter-electrode consistency based on the RSD of the response current or signal changes. Most reported results indicate that these sensors exhibit good preparation reproducibility on a laboratory scale. However, RSD data across batches are rarely reported. Practical implementation still faces challenges such as operational complexity, increased costs, and difficulties in quality control arising from multi-step drop-coating, nanomaterial synthesis, recognition layer immobilization, incubation, and washing. Future research should further standardize methods for evaluating stability and reproducibility, particularly by strengthening systematic investigations into batch-to-batch variations, stability during transportation and storage, and continuous detection performance on real samples, in order to enhance the feasibility of transitioning modified electrodes from laboratory preparation to mass production and field applications.

Given the above challenges, future research could focus on optimization in areas such as sample pretreatment, anti-contamination interfaces, selection of recognition modes, multi-target detection, intelligent data analysis, and standardization validation.

First, sample pretreatment strategies should be optimized to meet the detection requirements of electrochemical sensors. Proteins, lipids, salts, and pigments in aquatic products not only affect the extraction efficiency of targets but also interfere with electron transfer at the electrode interface and the stability of the recognition elements. Sample preparation should not merely aim for high spiked recovery rates but should systematically evaluate the compatibility between the extract and the sensing interface. For antibiotics such as tetracyclines that readily form complexes with metal ions, special attention should be paid to controlling pH, buffer systems, and the type of chelating agents. For targets that rely on direct electrochemical responses (e.g., chloramphenicol and nitrofurans), methods such as solid-phase extraction or modified QuEChERS should be employed to minimize interference from co-extracted substances on peak current and peak potential. For aptamer, antibody, and MIP-based sensors, the effects of residual solvents, salinity, and pH on the binding capacity of the recognition layer should be evaluated.

Second, the focus should shift from constructing high-response interfaces to developing stable, anti-contamination, and scalable sensor interfaces. Carbon-based materials, metal nanomaterials, MOFs/COFs, and conductive polymers have been widely used for signal amplification. However, they may still face issues such as interface contamination, signal drift, and inter-batch variations in complex aquatic matrices. Future work could draw on anti-contamination interface designs from electrochemical sensing of complex biological samples and food matrices, further incorporating anti-contamination layers such as hydrogels, PEG and its derivatives, zwitterionic polymers, and peptides onto highly conductive/highly catalytic interfaces to reduce the nonspecific adsorption of proteins and lipids [134,135]. At the same time, differentiated evaluation criteria should be established for different electrode platforms: for traditional electrodes such as GCE, gold electrodes, and ITO, the focus should be on evaluating regenerative capacity and long-term stability. For disposable platforms such as SPE, paper-based electrodes, LIGE, and flexible electrodes, the focus should be on evaluating inter-batch consistency, storage stability, and quality control in large-scale production.

Third, appropriate detection modes should be selected based on the molecular structure and electrochemical behavior of the target antibiotics. For antibiotics with distinct electrochemical activity, the focus should be on optimizing the direct electrocatalytic interface, peak resolution, and resistance to matrix interference. For antibiotics with weak electrochemical responses, emphasis should be placed on the stable immobilization and signal amplification of recognition elements. Future research should not blindly pursue complex nanocomposite structures but should instead design interfaces around practical detection challenges. For classes with numerous structural analogs, priority should be given to improving selective recognition and peak resolution. For trace amounts of species, emphasis should be placed on improving the signal-to-noise ratio in the low-concentration range. For multi-residue detection, attention should be focused on signal cross-interference and response correction, rather than merely the simultaneous appearance of multiple electrochemical peaks.

Fourth, efforts should be made to advance multi-target detection platforms toward integration, calibratability, and verifiability. Multi-channel electrode arrays, ratiometric signals, built-in reference probes, and portable multi-channel readout systems represent important areas of development. However, issues such as peak overlap, signal crosstalk, cross-recognition, and matrix drift still need to be addressed. It is recommended to incorporate internal standard calibration, blank matrix correction, and multichannel cross-validation mechanisms during the sensor design phase and to clearly evaluate selectivity, response independence, and quantitative accuracy among different analytes.

Fifth, machine learning and multivariate data analysis can serve as potential auxiliary tools for the analysis of complex electrochemical signals. Currently, research on electrochemical sensors for antibiotic residues in aquatic products primarily relies on single indicators (such as peak current, peak potential, or impedance values) for quantitative analysis, with insufficient attention paid to issues caused by complex matrices (such as background drift, peak shape changes, and the overlap of multiple target peaks). In the future, based on standardized data acquisition, machine learning could be applied to feature extraction and pattern recognition of raw electrochemical curves to assist in background subtraction, peak overlap resolution, and signal deconvolution in complex matrices. Furthermore, matrix effect correction models could be established by incorporating sample type, pretreatment conditions, electrode batch, and detection parameters. It should be emphasized that the effective application of machine learning depends on high-quality datasets of sufficient scale, external validation samples, and reliable comparisons with standard methods. Overfitting models based on small samples of spiked data should be avoided.

Finally, validation using real samples and standard methods should be strengthened. Currently, most studies have reported detection limits, linear ranges, and spiked recovery rates and typically include some evaluation of interference resistance and storage stability. However, these validations are mostly focused on laboratory-spiked samples, and stability evaluations are often limited to short-term storage or signal retention under single conditions. Systematic validation of quantification limits, matrix effects, inter-batch reproducibility, naturally contaminated samples, different types of aquatic products, and different tissue sites remains relatively insufficient. Future research should build upon existing performance evaluations to further strengthen comparisons with standard methods such as HPLC or LC-MS/MS and establish a validation system that more closely mirrors real-world regulatory scenarios. Only by forming a closed-loop system that integrates sample preparation, interface design, data analysis, and methodological validation can electrochemical sensors reliably transition from laboratory research to rapid screening and on-site regulatory applications for antibiotic residues in aquatic products.

## 6. Conclusions

**Figure 8:**
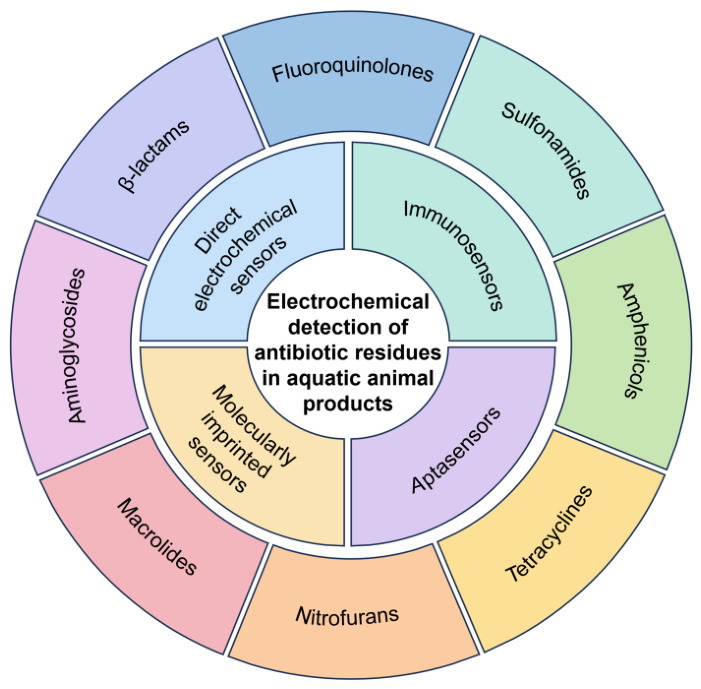
Schematic of the electrochemical detection of antibiotic residues in aquatic animals.

This paper provides a comprehensive overview of advances in the electrochemical detection of antibiotic residues in aquatic animals. It discusses eight classes of antibiotics: fluoroquinolones, sulfonamides, amphenicols, tetracyclines, nitrofurans, macrolides, aminoglycosides, and β-lactams, while also involving four types of sensors: direct electrochemical sensors, immunosensors, aptasensors, and molecularly imprinted sensors (Figure 8). Different classes of antibiotics exhibit significant differences in molecular structure, electrochemical activity, and recognition requirements. Antibiotics with intrinsic electrochemical activity, such as fluoroquinolones, nitrofurans, tetracyclines, and chloramphenicol, are more suitable for direct electrocatalytic detection, whereas targets with weaker electrochemical responses or higher selectivity requirements rely more heavily on recognition elements such as antibodies, aptamers, or MIPs to achieve selective detection.

In recent years, advancements in nanomaterial modification, composite interface construction, signal amplification, and portable platforms have significantly improved the sensitivity, selectivity, and analysis speed of electrochemical sensors in antibiotic residue detection. At the same time, this paper emphasizes the importance of sample pretreatment for aquatic product matrices. Matrix components such as proteins, lipids, salts, and endogenous small molecules may affect the release of target analytes, electron transfer at the electrode interface, and the stability of the recognition layer. Therefore, sample pretreatment and sensor interface design must be optimized in tandem to improve the reliability of detection in real samples.

In summary, electrochemical (bio)sensors offer advantages such as rapid response, low cost, ease of miniaturization, and suitability for on-site screening, making them an important complement to confirmatory methods such as HPLC and LC-MS/MS. However, their practical value depends not only on detection limits but also on their ability to resist interference in complex aquatic matrices, the consistency of electrode fabrication, the stability of recognition elements, and the level of validation with real samples. In the future, if efforts are made to further enhance anti-contamination interface design, standardize sample preparation, enable mass production of electrodes, implement multi-target signal correction, and conduct rigorous methodological validation, electrochemical sensing technology is expected to play a greater role in the rapid screening of antibiotic residues in aquatic products and on-site regulatory monitoring.

## Figures and Tables

**Table 1:** Summary of representative electrochemical detection methods for antibiotics in aquatic animals.

Antibiotic Class	Analyte	Sensor Type	Working Electrode	Analytical Method	LOD ^†^	Linear Range	Real Sample *	Ref.
Fluoroquinolones	ENR	direct electrocatalytic sensor	Ce_2_(MoO_4_)_3_/GCE	SWV	0.035 µM	0.1–12 µM	fish, shrimp, water	[34]
	ENR	direct electrocatalytic sensor	Cu/Cu_2_O@C_700_/NCs/GCE	DPV	7.73 × 10^−3^ ng mL^−1^	0.1–1000 ng mL^−1^	fish, shrimp	[35]
	ENR	MIP-based sensor	MWCNTs/GCE	DPV	0.9 pM	2.8 pM–28 µM	fish, shrimp, seawater	[32]
	ENR	MIP-based sensor	AuNP/NiFe-LDH/MWCNT/GCE	DPV	0.025 µM	0.05–100 µM	yellow croaker, crucian carp, shrimp, clam	[36]
	ENR	immunosensor	AuNPs/AuE	DPV	4.17 pg/mL	10^−6^ –10^2^ ng/mL	fish, chicken	[37]
	NOR + ENR	immunosensor	SPGE	SWV	NOR: 2.02 ng/mL ENR: 1.70 ng/mL	0.01–10 μg/mL	milk, honey, and fish	[38]
Sulfonamides	SM2	immunosensor	COOH-MWCNTs-Fe_3_O_4_-GO/GCE	DPV	0.003 ng/mL	0.01–100 ng/mL	crayfish	[39]
	SDM	aptasensor	AuNP/rGO/PGE	EIS	3.7 × 10^−16^ M	1.0 × 10^−15^–1.0 × 10^−5^ M	fish, chicken, beef	[40]
	SM2	MIP-based sensor	v-COF@SWCNTs-COOH/SPCE	DPV	0.21 nM	0.01–31.25 nM	fish, milk	[33]
	SDZ	MIP-based sensor	CuInS_2_/ZnS/GCE	DPV	2.1 nM	0.05–12.5 µM & 12.5–25 µM	fish, beef, milk	[41]
	SMX	direct electrocatalytic sensor	MIL-101(Fe)@N-Cu-MOF/GCE	DPV	0.0037 µM	0.01–150 µM	fish, shrimp, milk, serum, urine	[42]
	SN, SDZ, SM2, and SMX	direct electrocatalytic sensor	AuNSs/LIPG	DPV & CV	SN: 0.035 µM; SDZ: 0.062 µM; SM2: 0.055 µM; SMX: 0.048 µM	0.4–100 µM	fish, shrimp	[43]
Amphenicols	CAP	direct electrocatalytic sensor	β-CD/CuO/GCE	SWV	0.05 µmol L^−1^	0.1–20 µmol L^−1^ & 20–500 µmol L^−1^	chicken, duck, fish, milk	[44]
	CAP	direct electrocatalytic sensor	AgNPs/SF-GR/GCE	DPSV	0.01 µM	0.02–20.0 µM	shrimp	[45]
	CAP & NFT	direct electrocatalytic sensor	FeCo@S,N-CNTs/SPE	DPV	CAP: 20.0 nM; NFT: 3.5 nM	CAP: 0.05–320.0 µM; NFT: 0.01–1.0 µM & 1.0–75.0 µM	milk, honey, eggs, fish, chicken, pork	[46]
	CAP	immunosensor	HGNs/CS/GCE	DPV	0.06 ng mL^−1^	0.1–1000 ng mL^−1^	beef, fish, pork	[47]
	CAP	aptasensor	AuNPs/CS-GA/PB/SPCE	LSV	0.65 ng L^−1^	1.0–6.0 × 10^6^ ng L^−1^	milk, shrimp pond water, and shrimp meat	[48]
	FF	aptasensor	Au@CoMnN-Cs/AuE	DPV	5.28 × 10^−4^ ng mL^−1^	1 × 10^−3^–1 × 10^3^ ng mL^−1^	milk, egg, shrimp	[49]
Tetracyclines	CTC	MIP-based sensor	Au-PtRu/V_2_CT_x_/GCE	DPV	0.16 µM	1–120 µM	fish, chicken, pork	[50]
	TC	MIP-based sensor	MIOPPy-AuNP/SPCE	DPV	0.65 µmol/L	1–20 µmol/L	shrimp	[51]
	TC	aptasensor	Ni-HHTP@CS/SPCE	SWV	1.9 pM	10 pM–1.0 µM	*Ctenopharyngodon idella*, milk	[52]
	TC	aptasensor	PAN@Cu-BTC/SPE	SWV	0.32 pM	10 pM–1.0 µM	*Ctenopharyngodon idella*	[53]
	TC	aptasensor	AuE	DPV	28.4 pM	0.0001–1.0 µM	fish	[54]
	OTC	aptasensor	AuNPs/CMK-3/GCE	*i*–*t*	8.13 fg mL^−1^	100 fg mL^−1^–100 ng mL^−1^	honey, milk, chicken, fish, beef	[55]
	OTC	immunosensor	SPGE	DPV	0.33 ng mL^−1^	1–200 ng mL^−1^	milk, honey, shrimp	[56]
Nitrofurans	FLD	direct electrocatalytic sensor	ZnO-ZnCo_2_O_4_/GCE	DPV	1.46 nM	0.01–4.68 µM & 15–100 µM	fish	[57]
	NFT and FZD	direct electrocatalytic sensor	PEO/Au-Ag-ANCCs/ZnO-NPs/CPE	SWV	NFT: 0.26 pM FZD: 0.023 pM	NFT: 1.0 pM–250 µM FZD: 0.9 nM–360 µM	fish, chicken, honey, milk, wastewater	[58]
	NFT, FZD, NFZ and FLD	direct electrocatalytic sensor	CuNC/Ru@S-CN/LIGE	DPV	NFT: 0.98 nM; FZD: 0.25 nM; NFZ: 1.6 nM; FLD: 8.7 nM	NFT: 3.0 nM–15 µM; FZD: 0.80 nM–12 µM; NFZ: 5.0 nM–45 µM; FLD: 30 nM–18 µM	aquaculture sediment, aquaculture water, grass carp, *Penaeus orientalis*	[59]
	NFZ	direct electrocatalytic sensor	LIG-50 array	LSV	0.035 µM	0.2–8.0 µM	fish	[60]
	FZD	direct electrocatalytic sensor	MSFM	DPV	0.05034 µmol/L	0.1–100.0 µmol/L	fish	[61]
	FZD	MIP-based sensor	c-MWCNT-PMo_12_/GCE	CV	3.38 nM	0.006–0.09 & 0.09–0.6 µM	shrimp	[62]
Macrolides	AZM, ENR	direct electrocatalytic sensor	TA-Au-Ag-ANpM/Fe-dop-PANI/NiO-NPs/GCE	SWV	AZM: 0.053 pM; ENR: 0.013 pM	AZM: 0.8 pM–250 µM; ENR: 0.1 pM–550 µM	chicken meat, egg, fish, river, and lake water sample	[63]
Aminoglycosides	KANA; CAP	aptasensor	GCE	SWV	KANA: 35 fM; CAP: 21 fM	1 × 10^−4^ to 50 nM	milk, fish	[64]
	KANA; AMP	aptasensor	GCE	SWV	KANA: 18 fM; AMP: 15 fM	0.05 pM–50 nM	milk, fish	[65]
	Hygromycin B	MIP-based sensor	Cu-MOF/Ti_3_C_2_T_x_/AuE	CV	1.92 nM	5 nM–5 µM	fish, pork, chicken	[66]
	TOB	aptasensor	AB/PEI-AuNPs/GCE	DPV	0.19 fg mL^−1^	1.0 × 10^−5^–1.0 × 10^2^ ng mL^−1^	fish, chicken, duck, milk, honey	[67]
	KANA	aptasensor	CC	DPV	64.7 fM	1 pM–1 µM	milk, fish, honey	[68]
β-lactams	Amoxi	immunosensor	L-CYST-VS_2_/ITO	DPV	0.5 pM	1 pM–100 µM	fish	[69]

Abbreviation: ENR: enrofloxacin; NCs: nitrogen-doped longan shell carbon; MWCNTs: multi-walled carbon nanotubes; AuE: gold electrode; NOR: norfloxacin; SPGE: screen-printed graphene electrode; SM2: sulfamethazine; SDM: sulfadimethoxine; PGE: pencil graphite electrode; v-COF@SWCNTs-COOH: vertically grown covalent organic framework on carboxylic single-walled carbon nanotubes; SDZ: sulfadiazine; SMX: sulfamethoxazole; SN: sulfanilamide; LIPG: laser-induced porous graphene; CAP: chloramphenicol; β-CD: β-cyclodextrin; SF-GR: sulfonated functionalized graphene; DPSV: differential pulse stripping voltammetry; NFT: nitrofurantoin; HGNs/CS: hollow gold nanospheres/chitosan composite; CS-GA: chitosan-glutaraldehyde copolymer; PB: Prussian blue; FF: florfenicol; CoMnN-Cs: MOF-derived Mn, N Co-doped Co-C nanomaterials; CTC: chlortetracycline; TC: tetracycline; MIOPPy: molecularly imprinted overoxidized polypyrrole; Ni-HHTP: MOF of Ni^2+^-2,3,6,7,10,11-hexahydroxytriphenylene; PAN: polyaniline; Cu-BTC: copper-1,3,5-benzenetricarboxylic acid; OTC: oxytetracycline; FLD: furaltadone; PEO: polyethylene oxide; Au-Ag-ANCCs: gold-silver alloy nanocoral clusters; ZnO-NPs: ZnO nanoparticles; CPE: carbon paste electrode; FZD: furazolidone; NFZ: nitrofurazone; CuNC: N-doped carbon-stabilized atomically dispersed Cu sites derived from Cu@ZIF-8; Ru@S-CN: S-doped g-C_3_N_4_ ultrathin nanosheets supported Ru; MSFM: mesoporous silica film microelectrode; LIG-50 array: the engraved graphene array at 50% laser power percentage; c-MWCNT: carboxylated multi-walled carbon nanotubes; PMo12: phosphomolybdic acid; AZM: azithromycin; TA-Au-Ag-ANpM: thermally annealed gold-silver alloy nanoporous matrices; Fe-dop-PANI: iron-doped polyaniline; NiO-NPs: nickel oxide nanoparticles; KANA: kanamycin; AMP: ampicillin; TOB: tobramycin; AB/PEI: acetylene black/polyethyleneimine; CC: carbon cloth; Amoxi: amoxicillin; L-CYST-VS_2_: L-cysteine capped VS_2_ quantum dots. ^†^: The LOD values listed in the table are primarily used to compare the analytical sensitivity of different electrochemical sensors. Actual LOD requirements should be determined in conjunction with the regulatory status of each analyte and the corresponding maximum residue limit (MRL). *: Real samples provided in the table refer to all samples involved in the study, not just aquatic animals.

**Table 2:** Common pretreatment strategies for detecting antibiotics in aquatic animal samples.

Antibiotic Class	Main Extraction Systems	Concentration, Redissolution, and Pre-analysis Adjustment	Pretreatment Characteristics
Fluoroquinolones	Ethanol/acetic acid; Ethyl acetate/ammonia; Hydrochloric acid; Formic acid/acetonitrile; Methanol/water/acetic acid.	(1) Centrifugation after extraction to collect the supernatant; (2) Nitrogen drying or rotary evaporation in some methods; (3) Reconstitution with PBS, acetate buffer, or supporting electrolyte, with pH adjustment according to the detection conditions.	(1) Organic solvents or acidic extraction systems are commonly used, with relatively simple workflows; (2) pH and supporting electrolyte conditions have a clear influence on electrochemical responses.
Sulfonamides	Acetonitrile; Ethyl acetate; Acetonitrile/PBS; PBS.	(1) Centrifugation after extraction is commonly used to collect the supernatant, with filtration when necessary; (2) Some methods involve nitrogen drying followed by reconstitution with water or buffer, whereas simplified protocols use direct dilution before detection.	(1) Acetonitrile extraction is commonly used; (2) Simplified protocols often rely on standard addition method or matrix tolerance of the sensing interfaces.
Amphenicols	Ethyl acetate; Ethyl acetate/ammonia; Ethanol; PBS.	(1) Simplified protocols commonly use centrifugation, filtration, or dilution before detection; (2) Some methods involve nitrogen drying or rotary evaporation followed by reconstitution with PBS; (3) Lipid-rich samples are often treated with anhydrous sodium sulfate for dehydration and n-hexane for defatting.	(1) Lipids and hydrophobic co-extractives require particular attention; (2) Some methods include dehydration, defatting, and reconstitution steps.
Tetracyclines	EDTA-McIlvaine buffer; Ethyl acetate/ammonia; EDTA/acidic buffer/acetonitrile; Methanol; Acetonitrile	(1) Centrifugation after extraction is commonly used to collect the supernatant, with filtration when necessary; (2) Some methods include SPE cleanup or nitrogen drying; (3) Samples are commonly diluted with PBS or reconstituted with methanol/buffer before detection.	(1) Metal chelation is a key concern, with EDTA-containing or acid/base-assisted extraction systems commonly used; (2) pH, chelation state, and tissue binding are critical pretreatment factors.
Nitrofurans	Acetonitrile/water; Acetonitrile; Trichloroacetic acid; Deep eutectic solvents.	(1) Centrifugation after extraction is commonly used to collect the supernatant, with filtration when necessary; (2) Some methods involve nitrogen drying for concentration; (3) Samples are reconstituted with diluted acetonitrile, buffer, or supporting electrolyte before detection.	(1) Diverse extraction systems are used, mainly acetonitrile-based organic solvents; (2) Deep eutectic solvents have also been reported.

Note: “/” in main extraction systems indicates a mixed solvent system or a composite system in which multiple solvents are used simultaneously for extraction.

## Data Availability

No new data were created or analyzed in this study.
